# Benzothiazolium Derivative-Capped Silica Nanocomposites for β-Amyloid Imaging *In Vivo*

**DOI:** 10.1021/acs.analchem.1c02289

**Published:** 2021-09-08

**Authors:** Lijun Ma, Shu Yang, Yufan Ma, Yuzhi Chen, Zhenguo Wang, Tony D. James, Xuefei Wang, Zhuo Wang

**Affiliations:** † State Key Laboratory of Chemical Resource Engineering, College of Chemistry, Beijing Advanced Innovation Center for Soft Matter Science and Engineering, Beijing University of Chemical Technology, Beijing 100029, China; ‡ School of Chemistry and Chemical Engineering, University of Chinese Academy of Sciences, Beijing 100049, China; § Department of Chemistry, University of Bath, Bath BA2 7AY, U.K.; ∥ Department of Pharmacy, Beijing Tiantan Hospital, Capital Medical University, Beijing 100070, China; ¶ School of Chemistry and Chemical Engineering, Henan Normal University, Xinxiang 453007, China

## Abstract

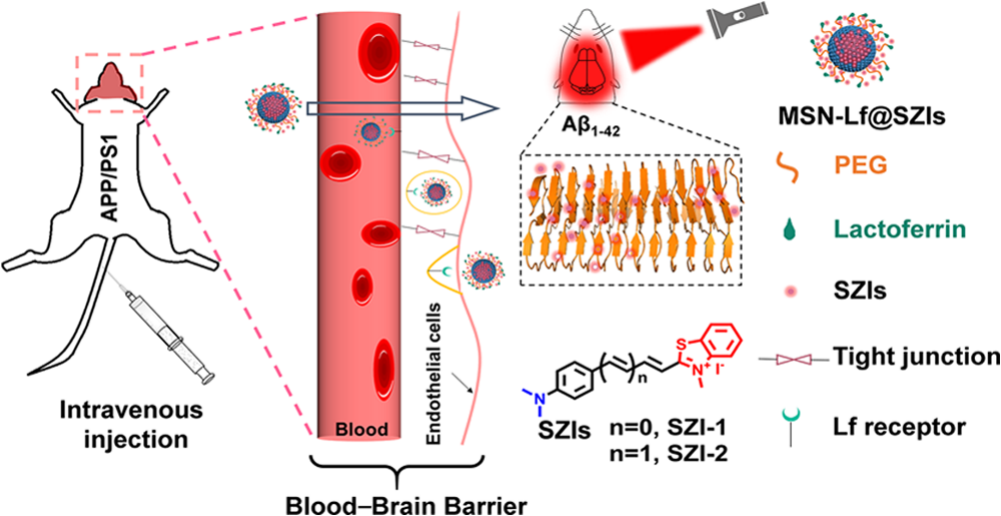

Alzheimer’s disease (AD) is a neurodegenerative disease, and β-amyloid (Aβ) is believed to be a causative factor in AD pathology. The abnormal deposition of Aβ is believed to be responsible for progression of AD. In order to facilitate the imaging of Aβ *in vivo*, suitable probe molecules with a near-infrared emission wavelength that can penetrate the blood–brain barrier (BBB) were utilized. The commercial fluorescent probe thioflavin-T (ThT) is used to image Aβ; however, because of its short emission wavelength and poor BBB penetration, ThT can only be used *in vitro*. With this research, based on ThT, we design three fluorescent probes (SZIs) having a longer emission wavelength in order to image Aβ aggregates. SZIs with different numbers of double bonds respond to Aβ aggregates. The SZIs have a structure similar to ThT, and as such, the SZIs are also unable to penetrate the BBB. To deal with the problem, we develop nanocomposites (MSN-Lf@SZIs) to deliver SZIs into the brain of AD mouse and image Aβ successfully. These new nanocomposites are able to deliver the dyes into the brain and facilitate Aβ imaging *in vivo*.

Alzheimer’s disease (AD) is a neurodegenerative disease, whose major pathological feature is the progressive loss of neuronal functions.^1^ β-amyloid (Aβ) is a proteolytic product of amyloid precursor protein (APP), which is a causative factor during AD pathology.^2^ Aβ includes many subtypes; the most common subtypes of Aβ in the human body are Aβ_1–40_ and Aβ_1–42_. Aβ_1–40_ and Aβ_1–42_ undergo similar transformation from the monomer, oligomer, to aggregate. Aβ_1–42_ has greater toxicity than Aβ_1–40_ and forms aggregates more easily due to the precipitation of Aβ, resulting in neurotoxicity. Therefore, Aβ_1–42_ is an important biomarker for the early diagnosis of AD.^3^

The current clinical methods for the diagnosis of AD are positron emission tomography and single-photon emission computed tomography.^4,5^ These two methods require suitable radionuclide-labeled probes as imaging agents and require time-consuming data acquisition and transfer of radioactive elements into the body. Compared with these two methods, optical imaging does not use radionuclides and can image the brain in real time.^6−18^ However, the brain has an innate barrier to maintain the homeostasis of the brain’s internal environment, which is called the blood–brain barrier (BBB). The BBB is connected by brain capillary endothelial cells (BCECS), pericytes, astrocytes, and neuronal cells.^19,20^ The tight junctions between the BCECS prevent paracellular transport of compounds from blood to the brain. Therefore, almost 100% of macromolecules and more than 98% of organic small molecules are blocked by the BBB.^21^ Aβ_1–42_ usually accumulates in the hippocampus and cortex of brains, and the imaging and diagnosis of Aβ_1–42_*in vivo* is difficult due to the existence of the BBB.^22^

The commercial probe thioflavin-T (ThT) is a standard probe, which is used for *in vitro* hippocampus and cortex staining to confirm the presence of Aβ_1–42_.^23^ As such, ThT is becoming the gold standard for the imaging and diagnosis of AD. ThT exhibits a specific interaction with Aβ_1–42_ aggregates. It binds to the Met35 and Gly27 residues of Aβ_1–40_ aggregates through hydrophobic interactions. As a result, the rotation between the benzene ring and benzothiazole is reduced and the quantum yield increases significantly due to the higher delocalization of the π electrons in the conjugated system induced by the twisted intramolecular charge-transfer (TICT) process.^24^ Although ThT has some advantages for the detection of Aβ_1–42_, it also has several inherent disadvantages such as poor BBB penetration (log *P* = −0.14) and short emission wavelength [λ_em_ = 490 nm in phosphate buffer saline (PBS)]. Generally, probes with log *P* values between 2 and 5 and molecular weights less than 500 Da coupled with near-infrared (NIR) emission wavelength are essential to penetrate the BBB and facilitate brain imaging *in vivo*.^25,26^ ThT has a low log *P* value (log *P* < 1) and as a result, does not cross the BBB and as a result, can only be used to image Aβ_1–42_*in vitro*.

From a scan of the literature, we discovered a series of molecules that have a similar structure to ThT, and we named these molecules SZIs.^27,28^ Compared with ThT, SZIs have an extended conjugated system with more double bonds in their structures, which may result in the emission wavelength of the TICT systems to be extended to the NIR region. As such, the SZIs have the potential for improving the spectral properties of ThT and facilitating the imaging Aβ_1–42_. Using calculations, SZIs have a higher affinity for Aβ_1–42_ aggregates. The theoretical data indicate that SZIs can bind with Aβ_1–42_ aggregates; however, the SZIs are still are unable to penetrate the BBB.

We set out to enable the BBB penetration ability of SZIs through the introduction of nanovectors. The new nanocomposites facilitate the transfer of the SZIs into the brain for imaging Aβ_1–42_*in vivo*. Receptor-mediate transportation is a promising method to assist in penetrating the BBB.^29,30^ Lactoferrin (Lf) receptors exist on the surface of brain glial cells, which can specifically bind to Lf and endocytose Lf into the brain.^31^ Herein, we report a new Lf-assisted nanocomposite (MSN-Lf@SZIs) to realize the *in vivo* imaging of Aβ_1–42_ in the brain. Our previous work supports a synergy of small molecules and nanocarriers to realize the targeting imaging and treatment *in vivo*.^32^ SZIs are similar in structure to ThT, and as such, the SZIs can bind strongly with Aβ_1–42_ aggregates. However, SZIs have low log *P* values and cannot penetrate the BBB. Therefore, using Lf surface-modified mesoporous silicon nanoparticles (MSNs), we are able to successfully transfer SZI-1 and SZI-2 across the BBB and image Aβ_1–42_ in the brain of mice. The fluorescence intensity of SZI-1 and SZI-2 is significantly increased by binding to Aβ_1–42_. *In vitro* and *in vivo* experiments confirm that the nanocomposites of the MSN-Lf and SZIs are capable of imaging Aβ_1–42_ in the brain of the living mice. Our research provides a new strategy, enabling the delivery of probes using a BBB-penetrating nanocomposite to realize Aβ_1–42_ imaging suitable for the auxiliary diagnosis of neurodegenerative diseases.

## Experimental Section

### Reagent, Materials, and Animals

All the reagents were commercial products and used without further purification unless otherwise stated. All the experimental water was deionized (DI). 4-(Dimethylamino)benzaldehyde, tetraethyl orthosilicate (TEOS), potassium *tert*-butoxide (*t*-BuOK), (3-aminopropyl)triethoxysilane (APTES), *N*-hydroxy succinimide (NHS), *N*-(3-dimethylaminopropyl)-*N*′-ethyl carbodiimide hydrochloride (EDC), iodomethane (CH_3_I), and 2,2,2-trifluoro-1-(trifluoromethyl)ethanol (HFIP) were purchased from Beijing Innochem Technology Co., Ltd. (1,3-Dioxolan-2-ylmethyl)triphenylphosphonium bromide was purchased from Shanghai Aladdin Biochemical Technology Co., Ltd. Hexadecyl trimethyl ammonium chloride (CTAC, 25% in water, v/v) was purchased from Tianjin Xi’ensi Biochemical Technology Co., Ltd. Triethanolamine (TEA) was purchased from Fuchen (Tianjin) Chemical Reagent Co., Ltd. 18-Crown-6 was purchased from Shanghai TCI Chemical Industry Development Co., Ltd. Poly(ethylene glycol) bis(carboxymethyl) ether (PEG, average *M*_n_ 600) and β-amyloid_(1–42)_human (Aβ_1–42_) were purchased from Shanghai Macklin Biochemical Technology Co., Ltd. Lf (CAS no. 112163-33-4) was purchased from Shanghai Ruiyong Biotechnology Co., Ltd. 2-Methylbenzothiazole was purchased from Alfa.

All the cell experimental reagents were sterile and used in a superclean bench to guarantee a sterile environment. High-glucose Dulbecco’s modified Eagle’s medium (DMEM), heat-inactivated fetal bovine serum (FBS), penicillin and streptomycin, and PBS (pH = 7.2–7.4, 0.01 M) were purchased from Hyclone. The human blood sample was provided by Beijing China-Japan Friendship Hospital. The 3-(4,5-dimethylthiazol-2-yl)-2,5-diphenyltetrazolium bromide (MTT) kit was purchased from KeyGEN BioTECH (Jiangsu, China).

Kunming (KM) mice (4–6 weeks, female), C57BL6 mice (10 months, female), and APP/PS1 (C57BL6, 10 months, female) were purchased from Beijing Huafukang Biotechnology Co., Ltd. All protocols requiring the use of animals were approved by the animal care committee of China-Japan Friendly Hospital. The approval number is zryhyy12-20-10-2.

### Instruments

^1^H and ^13^C NMR spectra were recorded on a Bruker Avance III (400 MHz, Germany) spectrometer in CDCl_3_ or DMSO-*d*_6_ solutions at room temperature (r.t.). Chemical shift (δ) is reported in parts per million downfield from tetramethylsilane; coupling constants (*J*) are reported in hertz (Hz), and the multiplicity is defined by s (singlet), d (doublet), t (triplet), or m (multiplet). UV–vis spectra were recorded on a UV–visible spectrophotometer (HITACHI, U-3900H, Japan). Fluorescence spectra were recorded on a fluorescence spectrophotometer (HITACHI, F-4600, Japan). Mass spectra results were obtained from the Beijing Mass Spectrometry Center, Institute of Chemistry, Chinese Academy of Sciences. Infrared spectra were recorded on a Fourier transform infrared spectrometer (iCAN9, Tianjin Energy Spectrum Technology Co., Ltd., China). A transmission electron microscope (HITACHI HT7700, Japan), laser scanning confocal microscope (Lecia SP8, Germany), and Zetasizer Nano (Malvern, Mastersizer 2000, UK) were used to characterize MSN and its modifications. Absolute fluorescence quantum yields were recorded on a full-featured steady-state transient spectrum analyzer (Edinburgh instruments, FLS980, England). Dissociation constant (*K*_d_) and cell viability values were recorded on a microplate reader (PerkinElmer Enspire, USA). The fluorescence signals of mouse brains were captured by an IVIS Lumina IV system (PerkinElmer Enspire, USA). The Gaussian calculation was supported by the high-performance computing platform of the Beijing University of Chemical Technology (BUCT).

### Synthesis

#### (*E*)-3-(4-(Dimethylamino)phenyl)acrylaldehyde (1)

Under a nitrogen atmosphere, (1,3-dioxolan-2-ylmethyl)triphenylphosphonium bromide (1.0 g, 2.28 mmol, 2 equiv) and potassium *tert*-butoxide (*t*-BuOK, 0.5 g, 4.56 mmol, 4 equiv) were first dissolved in freshly distilled tetrahydrofuran (THF) solution (30 mL) at 0 °C for 30 min. After that, 4-(dimethylamino)benzaldehyde (0.1 g, 0.67 mmol, 1.18 equiv) dissolved in freshly distilled THF solution (2 mL) was added slowly using a syringe. After the addition was complete, the reaction mixture was transferred to room temperature and stirred for 24 h. Compound 1 (0.12 g, 68.6%) was obtained by column chromatography (petroleum ether/dichloromethane/ethyl acetate = 5:1:1, v/v) as a yellow solid. ^1^H NMR (400 MHz, CDCl_3_): δ 3.09 (s, 6H), δ 6.56–6.62 (dd, 1H, *J* = 8.0 Hz and *J* = 8.0 Hz), δ 6.79 (d, 2H, *J* = 8.0 Hz), δ 7.41 (d, 1H, *J* = 16.0 Hz), δ 7.50 (d, 2H, *J* = 8.0 Hz), δ 9.63 (d, 1H, *J* = 8.0 Hz). MS *m*/*z*: calcd for C_11_H_13_NO, 175.23; found, 175.

#### (2*E*,4*E*)-5-(4-(Dimethylamino)phenyl)penta-2,4-dienal (2)

2 was synthesized using the same method as 1. A total of 0.054 g of 2 was obtained as a red solid with a yield of 40%. ^1^H NMR (400 MHz, CDCl_3_): δ 3.05 (s, 6H), δ 6.17–6.23 (dd, 1H, *J* = 8.0 Hz and *J* = 8.0 Hz), δ 6.70 (d, 2H, *J* = 8.0 Hz), δ 6.81–6.87 (dd, 1H, *J* = 12.0 Hz and *J* = 8.0 Hz), δ 6.97 (d, 1H, *J* = 16.0 Hz), δ 7.24–7.31 (d, 1H, *J* = 12.0 Hz and *J* = 8.0 Hz), δ 7.43 (d, 2H, *J* = 8.0 Hz), δ 9.58 (d, 1H, *J* = 8.0 Hz). MS *m*/*z*: calcd for C_13_H_15_NO, 201.12; found, 201.

#### 2,3-Dimethylbenzo[*d*]thiazol-3-ium Iodide (3)

To a solution of CH_3_CN (60 mL) were added 2-methyl benzothiazole (1.0 g, 6.7 mmol, 1 equiv) and CH_3_I (0.46 mL, 7.37 mmol, 1.1 equiv), and the mixture was refluxed at 82 °C for 24 h. An amount of white crystals precipitated and were collected by filtration and washed with CH_2_Cl_2_ to obtain 3 (1.8 g, 97%). ^1^H NMR (400 MHz, DMSO-*d*_6_): δ 8.44 (d, *J* = 8.1 Hz, 1H), 8.30 (d, *J* = 8.5 Hz, 1H), 7.91 (t, *J* = 8.4 Hz, 1H), 7.82 (t, *J* = 8.0 Hz, 1H), 4.21 (s, 3H), 3.18 (s, 3H). ^13^C NMR (400 MHz, DMSO-*d*_6_): δ 177.25, 141.56, 129.23, 128.68, 128.07, 124.46, 116.74, 36.14, 17.04.

#### (*E*)-2-(4-(Dimethylamino)styryl)-3-methylbenzo[*d*]thiazol-3-ium Iodide (SZI-1)

To a solution of CH_3_OH (30 mL) were added 4-(dimethylamino)benzaldehyde (0.0745 g, 0.5 mmol, 1 equiv) and 3 (0.1746 g, 0.6 mmol, 1.2 equiv), followed by the addition of a catalytic amount of pyridine; the resulting mixture was refluxed for 12 h. The reaction mixture was allowed to cool slowly to room temperature, and a reddish-brown solid was filtered off, washed with cold methanol, then with diethyl ether, and then recrystallized from methanol to afford compound SZI-1 (105.5 mg, yield 40%) as a red solid powder. ^1^H NMR (400 MHz, DMSO-*d*_6_): δ 8.31 (d, *J* = 7.9 Hz, 1H), 8.14–8.05 (m, 2H), 7.93 (d, *J* = 8.9 Hz, 2H), 7.80 (t, *J* = 7.6 Hz, 1H), 7.73–7.60 (m, 2H), 6.86 (d, *J* = 8.9 Hz, 2H), 4.24 (s, 3H), 3.13 (s, 6H). HRMS *m*/*z*: calcd for C_18_H_19_N_2_S, 295.13; found, 295.126346.

#### 2-((1*E*,3*E*)-4-(4-(Dimethylamino)phenyl)buta-1,3-dien-1-yl)-3-methylbenzo[*d*]thiazol-3-ium Iodide (SZI-2)

The synthesis of SZI-2 was similar to that of SZI-1. ^1^H NMR (400 MHz, methanol-*d*_4_): δ 8.12 (d, *J* = 8.0 Hz, 1H), 8.03–7.97 (m, 2H), 7.79 (t, *J* = 7.8 Hz, 1H), 7.68 (t, *J* = 7.7 Hz, 1H), 7.56 (d, *J* = 8.9 Hz, 2H), 7.37 (d, *J* = 14.9 Hz, 1H), 7.18 (dd, *J* = 14.8, 10.6 Hz, 2H), 6.77 (d, *J* = 8.9 Hz, 2H), 4.18 (s, 3H), 3.08 (s, 6H). HRMS (ESI) *m*/*z*: calcd for C_20_H_21_N_2_S, 321.14; found, 321.141996.

### Synthesis of MSN

To 20 mL of DI water (95 °C) was added CTAC (2.0 g, 25% in water) and TEA (0.8 g), and the mixture was stirred at 95 °C for 1 h under 400 rpm in a water bath. Then, 1.5 mL of TEOS was added slowly under 200 μL/min using an injection pump. After the addition of TEOS was complete, the mixture was continued to react for 1 h under the same condition. MSN was collected by centrifugation and washed with ethanol several times to remove CTAC. To remove CTAC completely, the MSN was dispersed into a mixture of ethanol/HCl (10:1, v/v) and refluxed for 12 h. The residual CTAC was monitored by a FTIR spectrometer.

### Synthesis of MSN-NH_2_

MSN-NH_2_ was synthesized by treatment with APTES. MSN (50 mg) was dispersed in 50 mL of ethanol and then refluxed for 4 h, followed by the addition of APTES (100 μL). When the reaction was finished, the mixture was centrifugated to remove excess APTES and washed with water several times to obtain MSN-NH_2_.

### Synthesis of MSN-PEG

MSN-PEG was synthesized by an amidation reaction. Bis-carboxy-polyethylene glycol (PEG, 0.1 g, 1 equiv) was first added into 20 mL of PBS solution, followed by the addition of EDC (0.032 g, 1 equiv) and NHS (0.0192 g, 1 equiv). The mixture was stirred at 0 °C for 30 min to activate the carboxyl of PEG. After that, a solution of MSN-NH_2_ (5 mg) was added and the resulting mixture was stirred at 4 °C overnight. MSN-PEG was collected by centrifugation and washed with PBS several times to remove excess PEG.

### Synthesis of MSN-Lf

To a solution of 10 mL of PBS (containing 5 mg of MSN-PEG) were added EDC (0.03 g) and NHS (0.02 g); the resulting mixture was stirred at 0 °C for 30 min to activate carboxyl. Then, Lf (0.1 g dissolved in 100 μL of ethanol) was added, and the mixture was stirred at 0 °C overnight. MSN-Lf was collected by centrifugation and washed with PBS several times to remove excess EDC, NHS, and Lf.

### Selectivity Experiment

Various ions, amino acids, and Aβ aggregates were prepared as a 20 μM solution, and SZIs were diluted in dimethyl sulfoxide (DMSO) at a concentration of 1 mg/mL. A total of 100 μL of ions, amino acids, and Aβ aggregate solutions were added to a black 96-well plate, and each concentration was prepared in triplicate. Then, 0.2 μL of SZI solution was added to each well; the black 96-well plate was placed in a shaker and shaken at 100 rpm for 30 min. A microplate reader was used to measure the fluorescence intensity of each well.

### Photostability Experiment

The DMSO solution of SZIs (1 mg/mL) was diluted to an appropriate concentration (0.25 μg/mL for SZI-1, 0.5 μg/ml for SZI-2, 50% propylene glycol/50% PBS, v/v). The SZI solution was placed under a white light source (15 mV) for continuous irradiation for different minutes, and the fluorescence intensity of SZIs was tested at different time points (0, 5, 10, 15, 20, 25, and 30 min).

### Cytotoxicity Experiment

The cell used for the cytotoxicity test was bEnd.3, purchased from the Chinese National Infrastructure of Cell Line Resource. The experiment was divided into two parts, cell culture and cytotoxicity test. The bEnd.3 cells were first cultured in cell culture fluid (high-glucose DMEM/heat-inactivated FBS/penicillin and streptomycin = 100:10:1, v/v) under 5% CO_2_ at 37 °C. After a period of incubation, bEnd.3 was transferred into a sterile 96-well plate (1 × 10^4^ cells per well) and cultured for 24 h under 5% CO_2_ at 37 °C. After that, the cell culture fluid was removed, and gradient concentrations of SZIs or MSN-Lf@SZIs in the cell culture fluid (0, 1, 2, 5, 10, and 15 μM for SZIs and MSN-Lf@SZIs) were added into the 96-well plate and incubated for another 24 h. After that, the gradient concentration of SZIs was removed and MTT (5× MTT was diluted into 1× MTT using dilution buffer, 50 μL per well) was added; the mixture was incubated at 37 °C for 4 h. The supernatant was removed, and formazan (150 μL per well) was added; the mixture was placed on a shaker to be mixed well. The cell viability was determined by a microplate reader at 490 nm.

### Hemolysis Experiment

Human blood (1 mL, provided by China-Japan Friendly Hospital, Beijing, China) was first diluted with 2 mL of PBS solution; the sample was centrifugated for 10 min (8000 rpm) and washed with PBS 5 times to separate red blood cells from serum. Finally, the red blood cells were dispersed in 10 mL of PBS solution, from which 0.2 mL was taken, and 0.8 mL of different reagents (PBS, DI water and 1, 2, 5, 10, and 15 μM of SZIs or MSN-Lf@SZIs) was added. After mixing evenly, the samples were allowed to stand still for 3 h at room temperature and centrifuged (12,000 rpm, 5 min) to determine hemolysis circumstances. The supernatant was removed, and its ultraviolet absorption was tested. The hemolysis rate (HR) of the red blood cells was calculated using the following formula
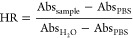
1

### Preparation of Aβ_1–42_ Aggregates

#### Pretreatment

To a solution of HFIP (600 μL) was added human Aβ_1–42_ (1 mg). After that, the solution was ultrasonically treated for 2 min to destroy the oligomer probably formed, followed by placing the mixture into an ice bath for 2 h. The solution was dispensed 20 μL per centrifuge tube, dried by nitrogen, and stored at −80 °C.

#### Aβ_1–42_ Aggregates

To a centrifuge tube (containing 33 μg of Aβ_1–42_) was added 90 μL of DI H_2_O and 1% hydroxylamine solution (10 μL); the solution was shaken on a shaker (300 rpm) at 37 °C for 72 h to form the Aβ_1–42_ aggregates. The Aβ_1–42_ aggregates were characterized by transmission electron microscopy (TEM).

### Fluorescence Enhancement Experiment

To a solution of probes (75 nM for SZI-1, SZI-2, and SZI-3) was added pretreated Aβ_1–42_ or bovine serum albumin (BSA) (30 μg per centrifuge tube). The mixture was incubated for 1 h with constant shaking (120 rpm). After that, the probe alone, probe and BSA, or Aβ_1–42_ fluorescence spectra was measured using a spectrophotometer (U-3900H, HITACHI, Japan, *b* = 10), of which the probe alone was used as the blank control. The fluorescence of PBS was also measured. The fold of fluorescence enhancement was calculated using the following formula: fold = the fluorescence intensity of the probe with Aβ_1–42_ or BSA/the fluorescence intensity of probe alone.

### Saturated Binding Assay

A solution containing a gradient concentration of probes and Aβ_1–42_ aggregates (30 μg/mL) in PBS were incubated at 37 °C with constant shaking (100 rpm) for 1 h. The solution of only probes (75 nM) in PBS (as a control) was also prepared in the same way. When the constant shaking was finished, the mixture and the control were transferred into a black 96-well plate. The fluorescence intensity was measured by a microplate reader. The obtained data were analyzed by GraphPad Prism 5.0 software, and the *K*_d_ value was calculated using nonlinear regression. The calculation method of *K*_d_ is shown in Table S2. All the samples were prepared in triplicate.

### *In Vivo* Fluorescence Imaging

The mice used for *in vivo* fluorescence imaging were APP/PS1 (10 months) and age-matched wild-type mice (WT, C57BL6). For *in vivo* fluorescence imaging, the APP/PS1 mice and wild mice were first head-shaved to reduce the effect of hair on fluorescence imaging. Before *in vivo* fluorescence imaging, the APP/PS1 and WT mice were first placed into an imaging box to obtain background signals. MSN-Lf@SZI-1 and MSN-Lf@SZI-2 were injected via the mouse tail vein, and the mice were transferred into an imaging box; the fluorescence signals of the brain were recorded at different time points on an IVIS Lumina IV system (PerkinElmer). The mice were anesthetized under 5% isoflurane gas and 1.0 L/min oxygen flow during the imaging process. The obtained data were analyzed with Living Image Software (Living software 4.5.5), and the region of interest (ROI) value was drawn around the brain region to obtain the fluorescence intensity of the brain region.

### *In Vitro* Fluorescence Staining

The paraffin-embedded blank sections were immersed in xylene for 5 min for deparaffinization and then washed with ethanol for 2 min and water for 5 min. The brain slices were incubated with 40 μL of 10 mg/mL ThT for 5 min and washed with 50% ethanol solution for 3 min. Next, the brain slices were incubated with 40 μL of 100 μM probes for 20 min. After absorbing the residual liquid with dust-free paper, the antifluorescence attenuating agent was added dropwise, and neutral gum was used for mounting. The slices were then placed under a laser confocal microscope (Lecia SP8) for imaging.

### BBB Uptake Test

To determine the BBB penetration rate of MSN-Lf@SZIs, we used liquid chromatography–mass spectrometry (LC–MS) to quantify the BBB penetration rate. Electrospray ionization mass spectroscopy (ESI–MS) was implemented with Shimadzu LCMS 2020. High-performance liquid chromatography analyses were made with a LC-20AD solvent delivery unit, SPD-M20A detector (Shimadzu), and Shim-pack GIST-HP column (3 μm C18, 2.1 mm × 150). A solution of MSN-Lf@SZI-1 (50% propylene glycol/50% PBS, 47.5 μg/mL, 125 μL) or MSN-Lf@SZI-2 (50% propylene glycol/50% PBS, 40 μg/mL, 125 μL) was injected into KM mice (28–30 g, 5–6 weeks, female) through intravenous injection. The mice were sacrificed at 5 min. Brain samples were removed, weighed, and homogenized with 1.0 mL of acetonitrile (LC–MS grade), and then, the leftover homogenate was extracted with 1.0 mL of acetonitrile twice; the total volume of acetonitrile was 3.0 mL. The extracted acetonitrile was filtered by a flashing nylon membrane (0.22 μm) to analyze by LC–MS. All the samples were detected in triplicate. The conditions used for LC–MS are listed in Table S9. Quantitative analysis was carried out from the peak area (the peak area was calculated using origin 8.0 software), and the brain uptake was presented by % injected dose per gram (% ID/g). The results of brain uptake were given as mean ± SD. The calculation formula is shown below.
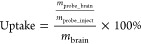
2where *m*_probe_inject_ means the quality of injection; *m*_brain_ means the wet weight of the brain. *m*_probe_brain_ means the quality of the probe in the brain, which was calculated according to the formula given below.
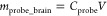
3where *C*_probe_ was calculated by the peak area according to the standard curve, and *V* means volume of the extract (3 mL).

## Results and Discussion

### Design, Synthesis, and Theoretical Calculation

**Figure 1:**
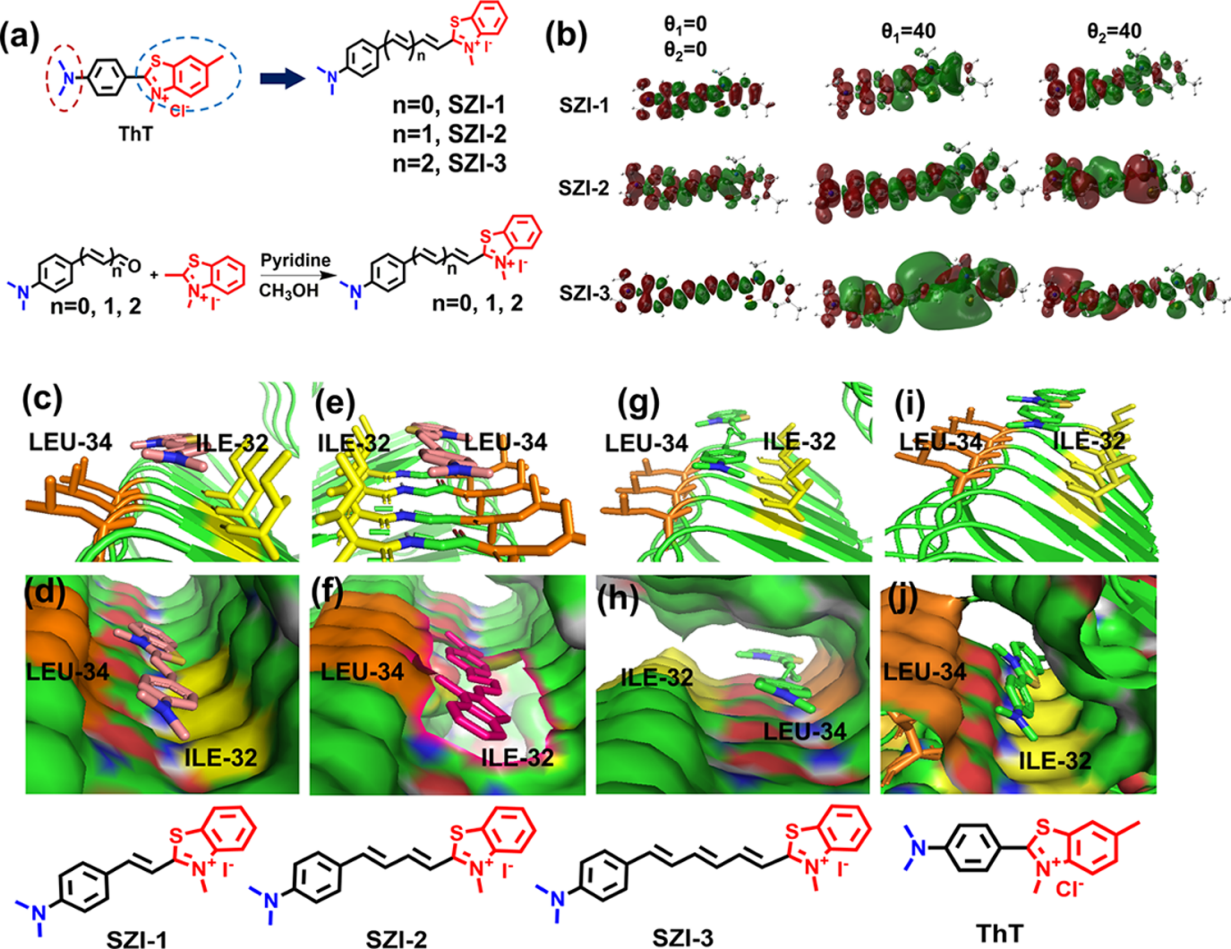
Design, synthesis, and theoretical calculations for SZIs. (a) Design and synthesis of SZIs. (b) Charge density difference (CDD) of SZIs with different dihedral angels (S_0_ state: θ = 0°and S_1_ state: θ = 40°). (c) Licorice form of SZI-1 in the Aβ_1–42_ aggregate model. (d) Molecular surface form of SZI-1 in the Aβ_1–42_ aggregate model. (e) Licorice form of SZI-2 in the Aβ_1–42_ aggregate model. (f) Molecular surface form of SZI-2 in the Aβ_1–42_ aggregate model. (g) Licorice form of SZI-3 in the Aβ_1–42_ aggregate model. (h) Molecular surface form of SZI-3 in the Aβ_1–42_ aggregate model. (i) Licorice form of ThT in the Aβ_1–42_ aggregate model. (j) Molecular surface form of ThT in the Aβ_1–42_ aggregate model.

SZI-1, SZI-2, and SZI-3 were designed according to the methods shown in Figure 1a. Aldehydes with a different number of double bonds were synthesized starting with the reaction of 4-(dimethylamino) benzaldehyde and (1,3-dioxolan-2-yl)methyl-triphenylphosphonium bromide with potassium *tert*-butoxide. This Wittig reaction was then repeated to generate different lengths of π-conjugated chains. The final SZI-1, SZI-2, and SZI-3 probes were then prepared using a nucleophilic addition reaction. A catalytic quantity of pyridine was used to deprotonate the 2,3-dimethylbenzo[*d*]thiazol-3-ium iodide, thereby activating nucleophilic addition with the aldehyde. The compounds (SZI-1, SZI-2, and SZI-3) were obtained in yields of 40, 26, and 15%, respectively. The number of double bonds for SZI-1, SZI-2, and SZI-3 were 1, 2, and 3, respectively. SZI-1, SZI-2, and SZI-3 were characterized by ^1^H NMR and HRMS (Figures S21–S32).

As shown in Figure 1a, the probes are composed of an electron-donating group (D) *N*,*N*-dimethylamino and an electron-withdrawing group (A) quaternary ammonium salt. The two groups are connected by a π-conjugated bridge, with typical D−π–A architecture in order to induce a TICT state. To further study the TICT in SZIs, the geometry optimization and excited-state energy calculation were performed using the method of B3LYP/6-311+(d,p). As shown in Figure 1b, the geometry optimization showed that all three molecules exhibited a planar configuration at the ground state. The profile of CDD between S_1_ and S_0_ gave direct presentation of intramolecular charge transfer (CT) under excitation, and no significant ICT was there at their lowest excited states. As shown in Figure S1d, if we set the plane of the alkene bridge as a reference, there were two typical dihedral angles (θ_1_ and θ_2_) between the alkene bridge and the planes of the other two aromatic moieties. The energy gap between S_0_ and S_1_ as a function of the individual angel had been estimated by modification of the two dihedral angles individually with an interval of 10°. In Figure S1, with the dihedral angles increasing, the energy gap decreased gradually. The wavelength difference between spectra and calculation should be attributed to the solvation effect. Here, for both molecules (SZI-1 and SZI-2), when θ_1_ was in the range of 40–50°, the transition energy decreased for the twist structure respective to that of the planar one, which was close to the spectral Stokes shift (70 nm and 120 nm, Table S2) of SZI-1 and SZI-2, respectively. For SZI-3, with the bridge length increasing, the dependence of transition energy on θ_1_ and θ_2_ was somewhat different; nevertheless, the same trend as SZI-1 and SZI-2 was observed. Therefore, it was predicted that a twisting process was probably involved in the excited-state relaxation. Although further increasing the twist angle resulted in lower transition energy, the corresponding decreased oscillator strengths (Table S3) implied the low contribution as the effective excited state. Further, their CDDs showed a significant ICT when θ_1_ = 40°, but that was absent in the case of the planar structure, demonstrating the presence of a TICT in SZIs.

The theoretical calculation results supported that the emission spectra of SZI-1 and SZI-2 exhibited a large Stokes shift caused mostly by a TICT effect. The red-shift emission wavelength of SZIs is particularly beneficial for bioimaging. Besides the emission spectra, we evaluated the interaction between SZIs and Aβ_1–42_ aggregates using theoretical calculations to evaluate the feasibility of using SZIs for Aβ_1–42_ imaging.

Since the structure of the SZIs is very similar to the structure of thioflavin-T (ThT), we assumed that the SZIs would also interact strongly with Aβ_1–42_ aggregates. As such, we evaluated the interaction between SZIs and Aβ_1–42_ aggregates using theoretical calculations. To date, for the mature Aβ_1–42_ aggregates, there are four kinds of high-resolution architectures that were observed in experiments, which were 2BEG, 2MXU, 5KK3, and 2NAO. Among these protein models, 2BEG was first reported. 2BEG was characterized by a U-shaped β-strand–turn−β-strand motif. Compared with 2BEG, another three models were characterized by a S-shaped triple-β motif.^33^ The 2MXU model exhibits a more complex three-dimensional amyloid structure.^34^ Besides, the 2MXU structure has a well-defined hydrophobic cleft and 12 β-strand filaments providing sufficient surface area for modeling the interactions with the probes.^35^ Therefore, the β-amyloid (Aβ_1–42_) aggregate protein model (PDB ID: 2MXU) was chosen to evaluate the interaction between Aβ_1–42_ aggregates and SZIs. The results were processed using Autodock Tools 1.5.6, PyMOL, and Python software, which are shown in the licorice form and molecular surface form. All the binding results are given in Tables S4–S7. The best binding energies were −6.72 kcal/mol (SZI-1), −7.37 kcal/mol (SZI-2), −8.27 kcal/mol (SZI-3), and −6.63 kcal/mol (ThT). These binding energies illustrated that SZIs could easily bind to the Aβ_1–42_ aggregates compared with ThT. The KLVFFA (Lys-Leu-Val-Phe-Phe-Ala) peptide is the core fragment of the Aβ_1–42_ protein. Therefore, the strong interaction between probes and KLVFFA is vital for the precise targeting of Aβ_1–42_ aggregates.^36^ As shown in Figure 1d,f,h, the molecular surface image clearly illustrates that SZIs can interact with LEU-34 (the core structure of KLVFFA) and ILE-32 residues and insert into the “hydrophobic channel” of the Aβ_1–42_ aggregates. Then, to determine the differences between the SZIs and ThT, we evaluated the interaction between ThT and Aβ_1–42_ aggregates. As shown in Figure 1i,j, ThT interacted with the LEU-34 and ILE-32 residues, which was the same as SZIs. The results inspired us to further evaluate the interactions between SZIs and Aβ_1–42_ aggregates *in vitro* and *in vivo*.

### Fluorescence Properties of SZIs and Their Response to Aβ_1–42_

We evaluated the optical properties of the SZIs in different solvents. In Table S2, the maxima emission wavelengths of the SZIs showed obvious red shift (from 593 to 803 nm in PBS solution) with an increasing number of π-conjugated units. The λ_ex_ and λ_em_ of the SZIs exhibited an obvious response to solvent polarity. The SZIs exhibit large Stokes shifts (64–285 nm from SZI-1 to SZI-3), which is particularly beneficial for bioimaging applications. The Stokes shift increases obviously with the number of π-conjugated units from SZI-1 to SZI-3. The SZIs display enhanced fluorescence in 1,2-propanediol (PDO) solution due to the reduced rotation of the chemical bonds being limited in more viscous solvents. Besides theoretical calculations, we further conducted experiments using solvents under various viscosity conditions to check the viscosity properties of SZIs to confirm the TICT. In Figure S2, both SZI-1 and SZI-2 exhibited enhanced fluorescence intensity when the viscosity of the solvents increased, which identified that SZI-1 and SZI-2 were TICT-based probes. The fluorescence of the SZIs correlates with the viscosity of the solvent. An increase in the number of π-conjugated units, especially SZI-3, resulted in a decrease of the fluorescence intensity, which mainly was due to the TICT effect and solvation effect. For SZI-3, the distance between the donor and acceptor was longer than SZI-1 and SZI-2. The electronic cloud distribution of SZI-3 was not obvious compared with SZI-1 and SZI-2 in the S_0_ state, which indicated that the ICT effect existing in SZI-3 was weaker than SZI-1 and SZI-2 (Figure 1b). In addition, the TICT present in SZI-3 and the influence of the solvation effect could cause the low quantum yield of SZI-3.^37^ Therefore, the fluorescence intensity of SZI-3 was much lower than the other SZIs and could not be used for biological experiments.

**Figure 2:**
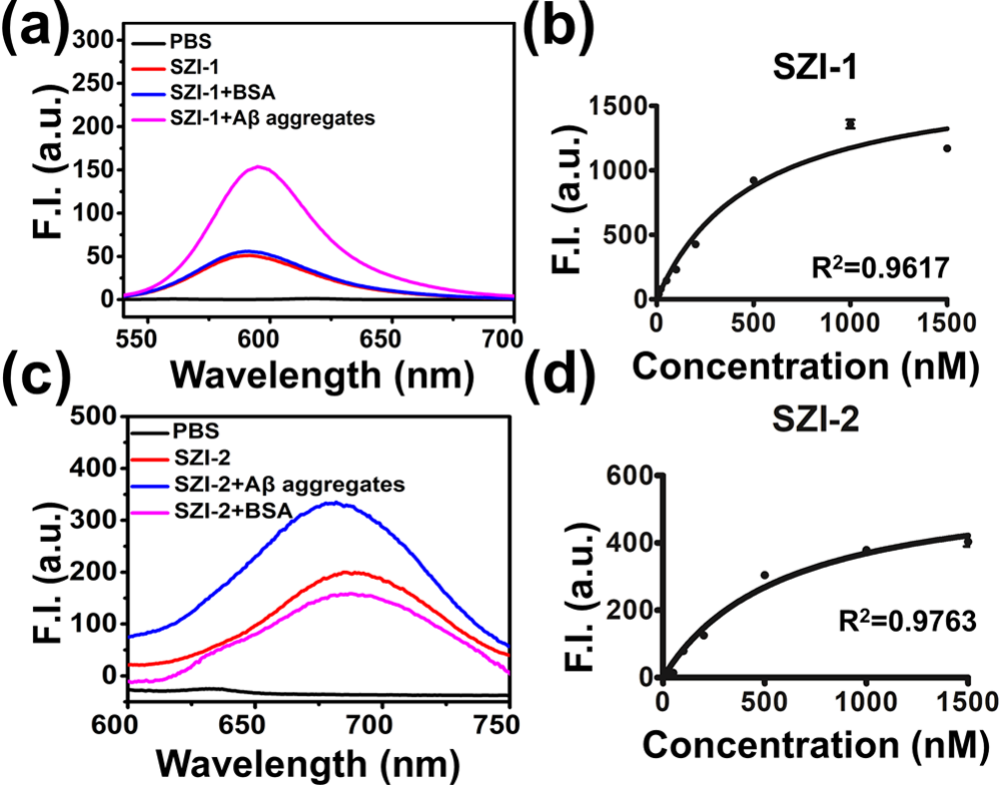
Optical properties and *K*_d_ of SZIs. (a) Fluorescence response curve of SZI-1 (50 nM in PBS) to Aβ_1–42_ aggregates (20 μg/mL in PBS, final concentration) or BSA (10 μg/mL, final concentration). (b) Saturated binding assay curve of SZI-1 to Aβ_1–42_ aggregates in different concentrations. All samples were tested in triplicate. (c) Fluorescence response curve of probe SZI-2 (50 nM in PBS) to Aβ_1–42_ aggregates (20 μg/mL in PBS, final concentration) or BSA (10 μg/mL, final concentration). (d) Saturated binding assay curve of SZI-2 to Aβ_1–42_ aggregates at different concentrations. All samples were tested in triplicate.

The fluorescence response of SZI-1 or SZI-2 to Aβ_1–42_ aggregates and BSA was tested in PBS solution. The formation of Aβ_1–42_ aggregates was confirmed by the TEM images (Figure S4). The morphology of the Aβ_1–42_ aggregates is filamentous. The fluorescence response of ThT with Aβ_1–42_ aggregates was tested to confirm the formation of the Aβ_1–42_ aggregates (Figure S5). The fluorescent response of ThT indicated that Aβ_1–42_ aggregates had been prepared successfully. We evaluated the interaction between SZI-1 and SZI-2 with Aβ_1–42_ aggregates. As shown in Figure 2a,b and Table S2, a threefold fluorescence enhancement was observed when SZI-1 interacted with Aβ_1–42_ aggregates. Besides, a red shift (∼5 nm) of emission wavelength was observed when SZI-1 interacted with Aβ_1–42_ aggregates. Compared with SZI-1, SZI-2 also showed interaction with Aβ_1–42_ aggregates, with a 1.7-fold fluorescence enhancement being obtained. We also observed a blue shift (∼17 nm) of emission wavelength when SZI-2 interacted with Aβ_1–42_ aggregates. These results indicated that SZI-1 and SZI-2 insert into the hydrophobic pocket of Aβ_1–42_ aggregates, which also illustrated that SZIs can specifically respond to Aβ_1–42_ aggregates. The *K*_d_ between SZI-1 and SZI-2 to Aβ_1–42_ aggregates were 507.6 ± 94.75 and 600.6 ± 94.82 nM, respectively (Table S2), which were smaller than ThT (*K*_d_ = 890 nM, Table S8). SZI-1 and SZI-2 showed better bonding affinity with Aβ_1–42_ aggregates.

We then imaged Aβ_1–42_ deposits using SZI-1 and SZI-2 *in vivo*. To determine whether SZI-1 or SZI-2 could penetrate the BBB, we chose KM mice (5–6 weeks) as the experimental animals. A solution of SZI-1 or SZI-2 (0.1 mg/kg, 50% propylene glycol/50% PBS, V/V) was injected into the KM mice by intravenous injection and used the IVIS Spectrum (PerkinElmer) instrument to image the mouse brain. Although SZIs have longer π-conjugated bridges than ThT, they still cannot penetrate the BBB in order to reach the brain for imaging. No fluorescence was observed in the brain region, which indicated that SZIs alone could not penetrate the BBB to image Aβ_1–42_ deposits *in vivo* (Figure S6b). The reason was mainly the low log *P* values of SZIs, making them unable to penetrate the BBB. Generally, log *P* (2 < log *P* < 5) is a requirement to guarantee BBB penetration. Therefore, we needed to find a delivery method capable of transporting the SZIs to the brain in order to alleviate this problem.

Therefore, in order to facilitate the BBB penetration by the SZIs, we introduced a surface-functionalized nanocomposite to aid SZI-1 and SZI-2 crossing the BBB. MSNs are known to cross the BBB; however, the MSNs alone show low penetration ability. To solve this problem, Lf was attached to the surface of the MSNs in order to enhance BBB penetration. Lf is a cationic ion-binding glycoprotein. Lf has excellent biocompatibility and high receptor-mediated transport efficiency. The Lf receptor exists in cerebrovascular endothelial cells. Through receptor-mediated endocytosis, Lf can interact with the Lf receptor and penetrate the BBB.

**Figure 3:**
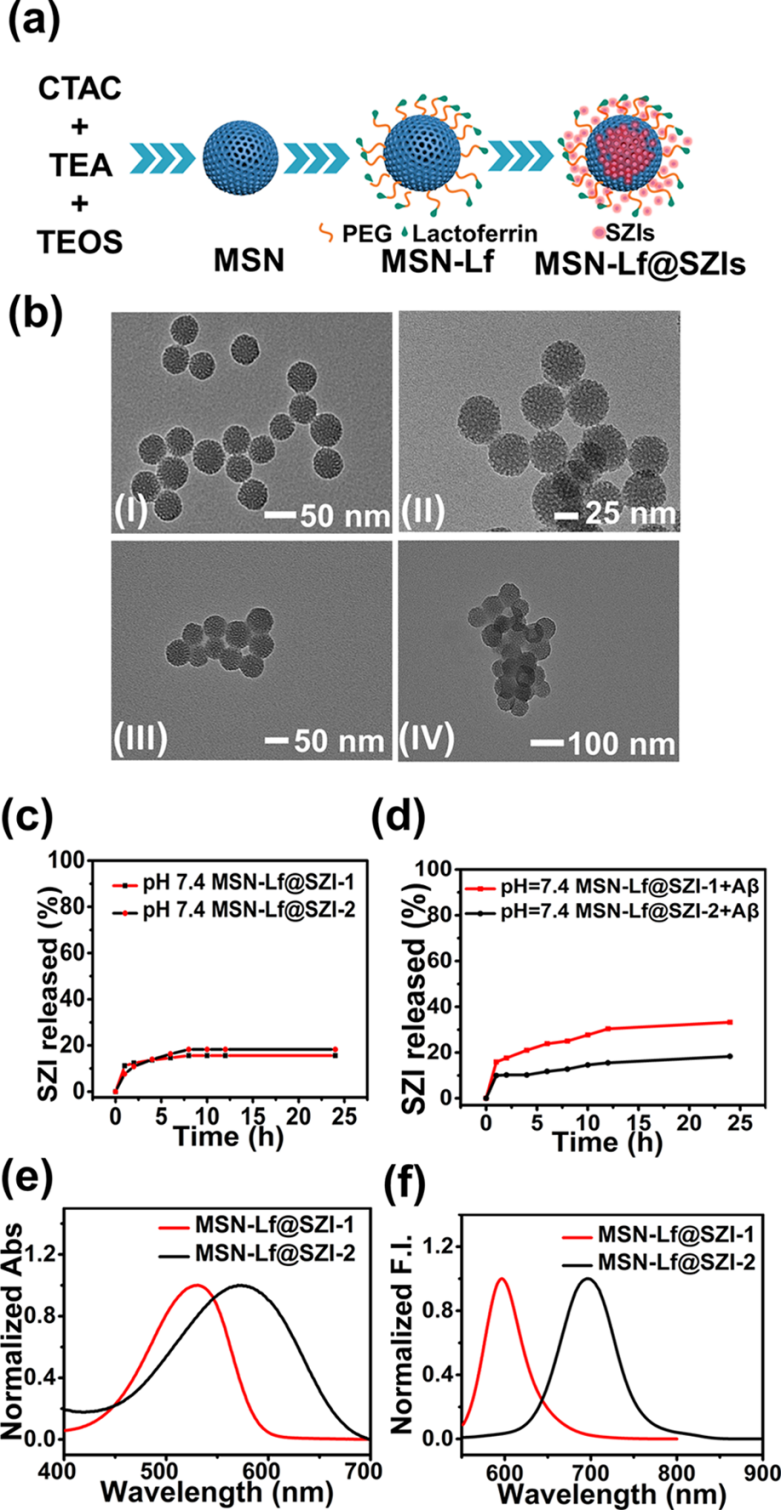
Properties of MSN. (a) Schematic diagram of MSN. (b) TEM images of MSN and functional MSN. (I) MSN, (II) MSN-NH_2_, (III) MSN-Lf, and (IV) MSN-Lf@SZIs. SZI release curve (pH = 7.4) of MSN-Lf@SZIs under experimental conditions (c) without Aβ_1–42_ aggregates and (d) with Aβ_1–42_ aggregates. The absorption curve (e) and emission curve (f) of MSN-Lf@SZIs (20 μg/mL in PBS).

We synthesized MSNs using CTAC as a template. The surface of the MSN was modified using amino groups to obtain MSN-NH_2_. MSN-NH_2_ was then reacted with COOH–PEG–COOH to introduce carboxyl groups to obtain MSN-PEG. Lf was attached to the MSN-PEG using an amidation reaction to generate MSN-Lf. MSN, MSN-NH_2_, and MSN-Lf displayed uniform morphology and good dispersion, with an average diameter of 46.80, 60.06, and 60.41 nm, respectively (Figures 3b and S8 and Table S1). The dynamic light scattering diameter was larger than that obtained from TEM due to the hydrated layers around the particle.^38^ The zeta potential of MSNs was determined to be −20.08 mV (Table S1), which indicated good stability. The zeta potential was found to be 17.64 mV (Table S1) when the amino groups were linked to the MSNs. The TEM image of MSN-NH_2_ (Figure S7) exhibited good dispersion of MSN-NH_2_. After modification with PEG, the zeta potential of the MSN-PEG changed slightly to 19.91 mV (Table S1). However, the zeta potential of MSN-Lf changed dramatically to 8.80 mV. Then, after loading the MSNs with the SZIs, the zeta potential of MSN-Lf@SZIs changed to 5.67 mV.

### Loading and Release of SZIs

MSN was used as a carrier to aid the BBB penetration of the SZIs. In order to realize the imaging of Aβ deposits *in vivo*, the loading and release rates of MSN-Lf@SZIs were evaluated. In order to simulate the *in vivo* environment, the release experiment was carried out in PBS solution (pH = 7.2–7.4). As shown in Figure 3c, a continuous release of SZIs was observed for 8 h in PBS. The release rates for MSN-Lf@SZI-1 and MSN-Lf@SZI-2 were 18 and 16%, respectively. After 8 h, SZIs were basically not released. The release process in the presence of Aβ_1–42_ aggregates was recorded. The release time was extended to 12 h and the total release of SZIs was higher with Aβ_1–42_ aggregates. When the release time reached 24 h, the total release of MSN-Lf@SZI-1 was about 33.22%.

To evaluate the optical properties of the MSN-Lf@SZIs, we evaluated the absorption wavelength (λ_ex_) and emission wavelength (λ_em_) for MSN-Lf@SZIs in PBS solution. As shown in Figure 3e,f, the λ_ex_ and λ_em_ of MSN-Lf@SZIs were similar to SZIs. However, the fluorescence intensity of MSN-Lf@SZIs was weaker than the SZIs. The main reason was that the SZIs were contained within the pores of the mesoporous materials and aggregated to some extent, resulting in fluorescence quenching (Figures S14 and S15).

**Figure 4:**
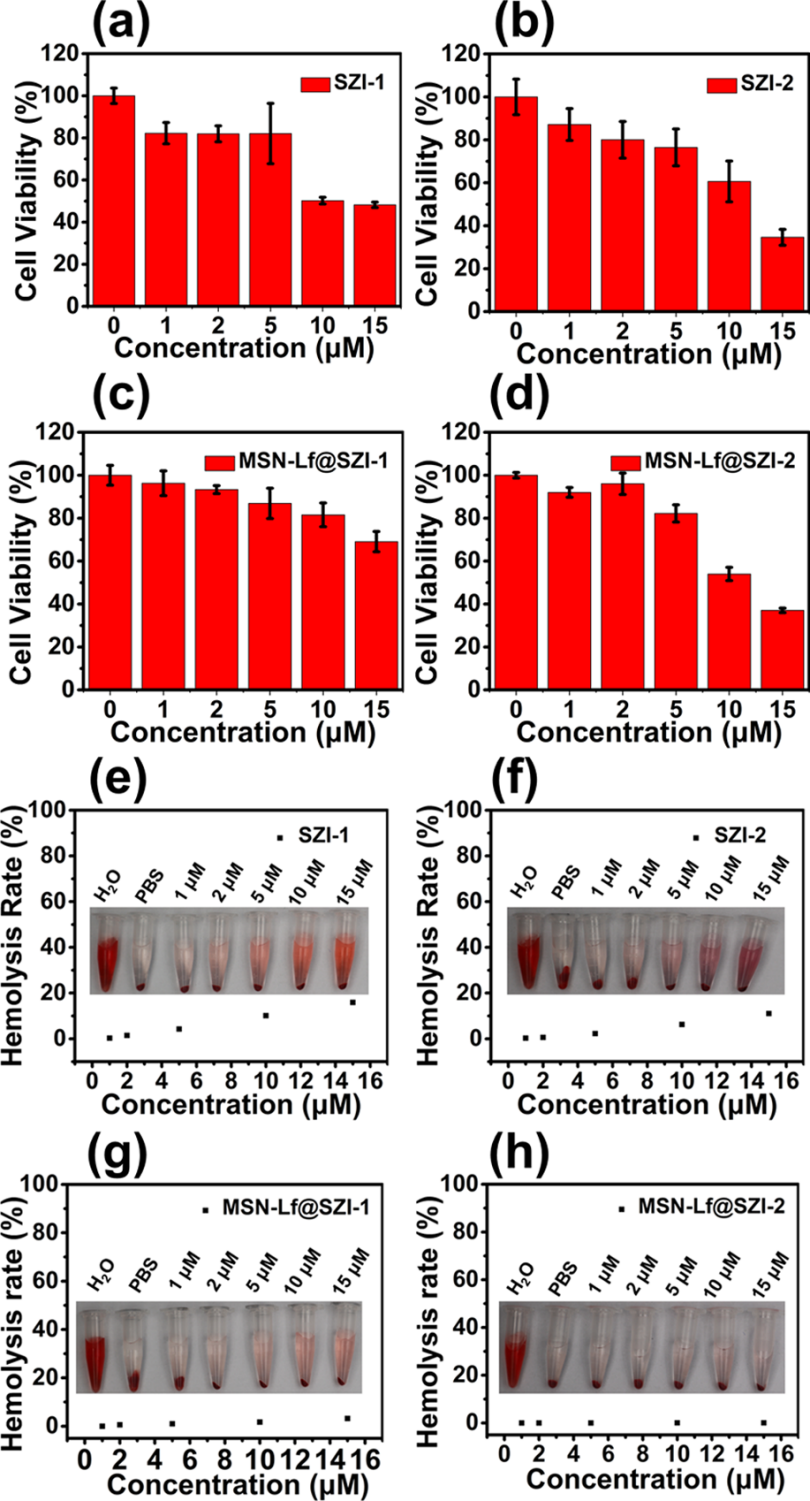
Biocompatibility of SZIs and MSN-Lf@SZIs. (a,b) Cytotoxicity of SZIs. (c,d) Cytotoxicity of MSN-Lf@SZIs. The cell used for the cytotoxicity experiment was bEnd.3. (e,f) HR of SZIs. (g,h) HR of MSN-Lf@SZIs.

The biocompatibility of SZIs was evaluated with bEnd.3 cells. The cytotoxicity of SZIs was measured by using a standard MTT assay. The cytotoxicity of SZIs exhibited relatively higher values (Figure 4a,b). We also evaluated the HR and found that the HR of SZIs was relatively high (Figure 4e,f). In contrast, the cytotoxicity and HR of MSN-Lf@SZIs were reduced to varying degrees (Figure 4c,d,g,h), which indicated that the existence of MSN-Lf was beneficial for subsequent biological experiments.

To guarantee that MSN-Lf@SZIs could cross the BBB, we evaluated the penetration of the BBB *in vivo*. As shown in Figure S6a,b, the probes SZI-1 and SZI-2 could not cross the BBB of KM mice, while both MSN-Lf@SZI-1 and MSN-Lf@SZI-2 could cross the BBB successfully. To quantify the BBB penetration rate, we used LC–MS to determine the concentration of SZIs in the brain. As shown in Tables S9 and S10, the BBB penetrations of MSN-Lf@SZI-1 and MSN-Lf@SZI-2 were 15.73 ± 8.51% ID/g and 7.09 ± 0.79% ID/g, respectively. The results indicated that both MSN-Lf@SZI-1 and MSN-Lf@SZI-2 could cross the BBB efficiently. Due to the presence of Lf receptors on the surface of BBB endothelial cells, nanoparticles can be transported across the BBB using receptor-mediated transport. Encouraged by these results, the imaging of transgenic mice (APP/PS1 double transgenic mice) by the MSN-Lf@SZIs nanocomposite was carried out in the following experiments.

APP/PS1 (AD) double transgenic mice can express a mutant human presenilin (DeltaE9) and human-mouse amyloid preprotein (APPswe) fusion, and the expression of both genes is initiated by the mouse prion protein promoter. The DeltaE9 mutation in the human presenilin gene is caused by the deletion of the ninth exon of the gene. This mutation can cause early-onset AD.^39^ APP/PS1(AD) double-transgenic mice aged 10 months will form Aβ deposits in the brain. Therefore, APP/PS1 double transgenic mice are an ideal animal model for evaluating the deposition of Aβ in the brain.

**Figure 5:**
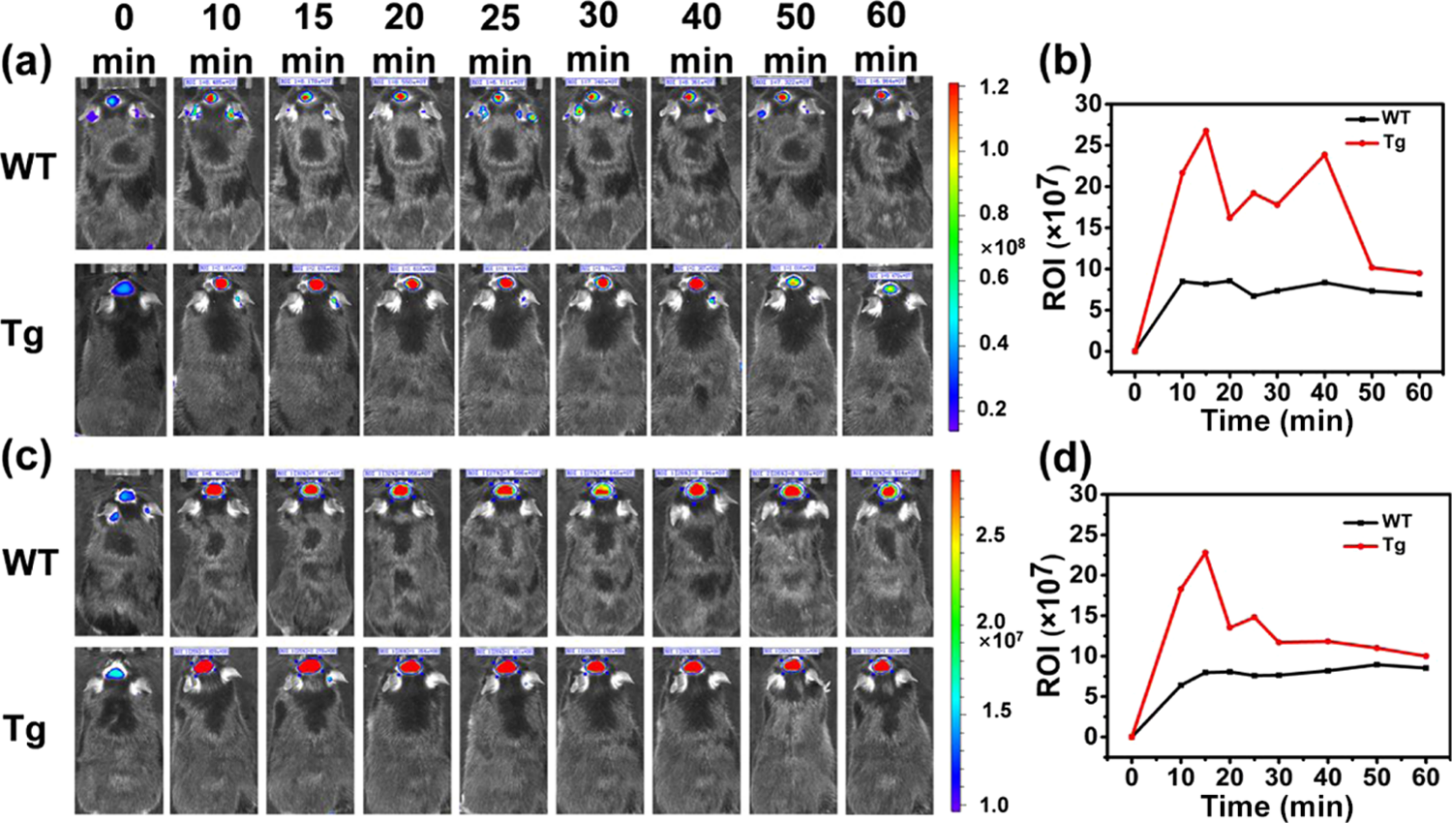
*In vivo* fluorescence imaging in the brain region of transgenic mice (Tg) and wild-type mice (WT) at different time points after intravenous injection of MSN-Lf@SZI-1 or MSN-Lf@SZI-2 (0.1 mg/kg in 50% PDO and 50% PBS). (a) The fluorescence signals of MSN-Lf@SZI-1 between the Tg and WT mice at different time points were obtained using the IVIS IV system. (b) Clearance curve of MSN-Lf@SZI-1 in the Tg and WT mice; the results were determined from the values of the ROI value. The unit of ROI is [(p/s/cm^2^/sr)/(μW/cm^2^)]. (c) The fluorescence signals of MSN-Lf@SZI-2 between the Tg and WT mice at different time points were obtained using the IVIS IV system. (d) Clearance curve of MSN-Lf@SZI-2 in the Tg and WT mice; the results were determined from the values of the ROI value. The unit of ROI is [(p/s/cm^2^/sr)/(μW/cm^2^)].

As shown in Figure 5a,c, the fluorescent images indicate that MSN-Lf@SZI-1 and MSN-Lf@SZI-2 could cross the BBB. The relative fluorescence intensity in the brain region decreased after 15 min by intravenous injection. The relative fluorescence intensity was obtained by choosing the ROI around the brain region to give a relative intensity value. The metabolism curve was plotted by the ROI *versus* time after intravenous injection (Figure 5b,d). The relative fluorescence intensity of transgenic-type (Tg) mice was slightly higher than that of WT mice until 60 min, which indicated that SZIs could specifically bind to Aβ plaques in the brain of Tg mice and imaged Aβ plaques for at least 1 h. In Figure 5b, the ROI value of the brain showed a fluctuating trend, which can be explained from the release curve and log *P* of the molecule. In Figure 3c,d, the SZI-1 release rate of MSN-Lf@SZI-1 in a solution of PBS containing Aβ_1–42_ aggregates was much higher than that in only PBS solution. The release rate of SZI-1 in Tg mice was higher than that in WT mice, so it could specifically bind to Aβ plaques. However, the clog *P* of SZI-1 was lower than 1 (clog *P* = 0.20), which indicated that SZI-1 was also very water-soluble, so SZI-1 could easily be metabolized by the brain. Since metabolism and release were carried out almost simultaneously, the ROI value of the mouse brain showed a fluctuating trend. When the release reaches the maximum, SZI-1 was slowly metabolized, and the ROI value gradually decreased. For MSN-Lf@SZI-2, it also showed a slightly fluctuating trend. Compared with SZI-1, the *K*_d_ value of SZI-2 (*K*_d_ = 600.6 ± 94.82 nM) was much higher than SZI-1 (*K*_d_ = 507.6 ± 94.75 nM), which indicated that the binding time of SZI-2 to Aβ plaques was not as long as that of SZI-1. Besides, the clog *P* value of SZI-2 (clog *P* = 1.26) was much higher than SZI-1, so SZI-2 could be metabolized from the brain slower than SZI-1. According to Figure 3d, the release rate of SZI-2 in the PBS solution containing Aβ_1–42_ aggregates did not increase significantly, so the release of SZI-2 in the mice may be slower. In Figures S14 and S15, the fluorescence intensity of MSN-Lf@SZI-2 is lower than MSN-Lf@SZI-1, which caused the fluctuation trend of SZI-2 to be not obvious. In summary, both MSN-Lf@SZI-1 and MSN-Lf@SZI-2 could image Aβ plaques for at least 1 h, which is longer than most molecular imaging agents.^40−44^

**Figure 6:**
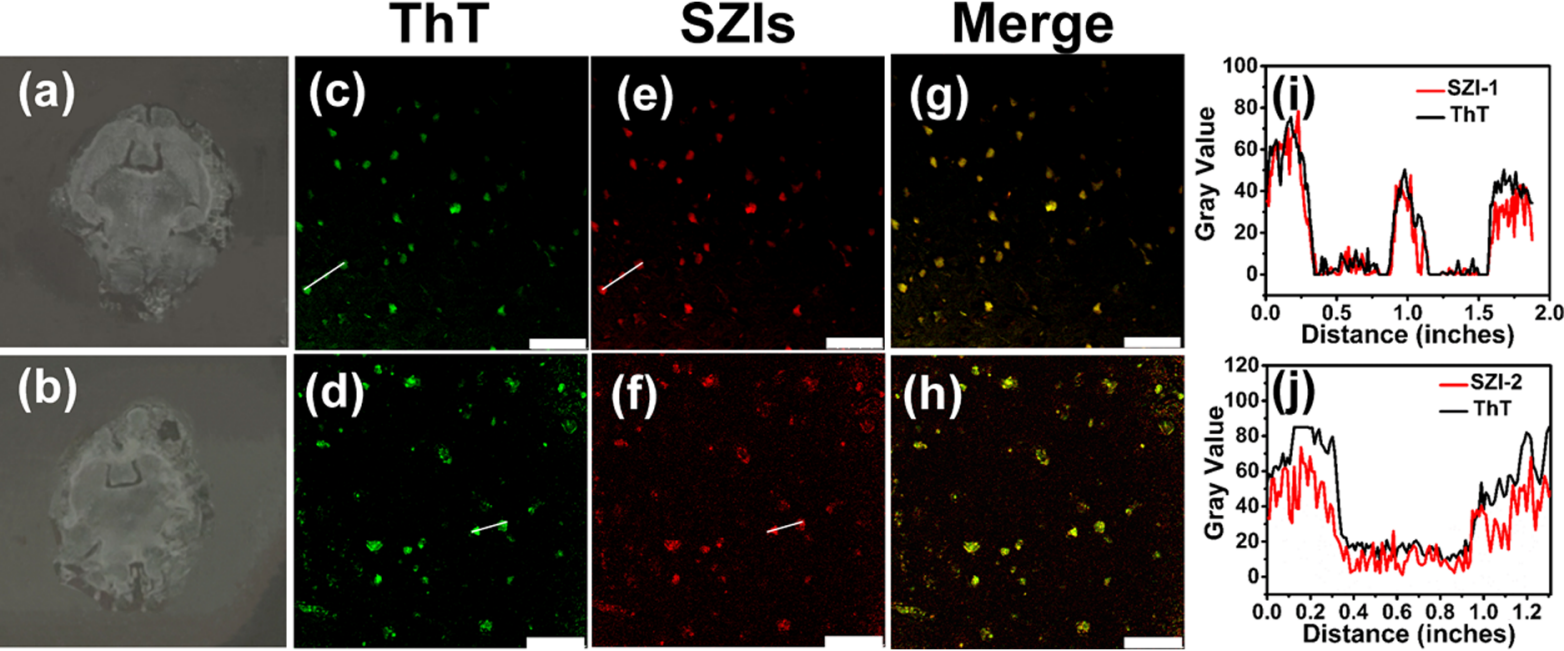
*In vitro* fluorescence staining results for brain slices from Tg mice (C57BL6, APP/PS1, 10 months old, female). (a,b) Image of the brain section before staining using ThT and SZI-1(SZI-2). (c,d) Image of the brain sections stained using ThT (Ex = 488 nm channel, 10×) as a control. (e,f) Staining results of SZI-1 (e, Ex = 552 nm channel, 10×) and SZI-2 (f, Ex = 552 nm channel, 10×). The homologous staining results (merged images) are shown in parts (g,h), respectively. The fifth column of images (i,j) represents the intensity profile of linear ROIs. The scale bar is 75 μm (c,e,g) and 50 μm (d,f,h).

To further confirm the binding of SZI-1 (SZI-2) to Aβ deposits, the fluorescence staining experiments were performed on the brain sections of Tg mice (10 months old, female). A confocal laser scanning microscope was used to image the brain sections stained using SZI-1 and SZI-2. As shown in Figure 6c,e,g, the fluorescence images of SZI-1 (Ex = 552 nm channel) displayed relatively good imaging for Aβ deposits. The same section was further stained using ThT (Ex = 488 nm channel) as a control. The spots obtained for SZI-1 merged well with those for ThT (Figure 6i). The sections stained using SZI-2 displayed similar results to those obtained with SZI-1(Figure 6d,f,j). These results clearly illustrated that SZI-1 and SZI-2 exhibit good imaging ability for Aβ deposits.

## Conclusions

We have developed two new composites for imaging of Aβ aggregates. SZIs have an extended conjugated structure when compared with ThT and exhibit a red shift of the emission wavelength. We evaluated the fluorescence response between SZIs and Aβ aggregates. The enhanced fluorescence response to Aβ aggregates is due to TICT process of SZIs. The binding affinity of SZI-1 for Aβ aggregates was 507.6 ± 94.75 nM, and the binding affinity of SZI-2 for Aβ aggregates was 600.6 ± 94.82 nM. Molecular docking studies indicated that the fluorescence enhancement with Aβ aggregates by SZI-1 or SZI-2 was mainly because they were inserted into the hydrophobic cavity of the Aβ aggregates, restricting the rotation of the probe and enhancing the fluorescence output. The nanocomposites (MSN-Lf@SZIs) were developed to facilitate the *in vivo* entry of the SZIs into the brain. MSN-Lf@SZIs can image Aβ_1–42_ of a transgenic AD mouse brain for a longer time (>60 min) than most of the reported molecular probes in the brain. MSN-Lf@SZIs present better biocompatibility than SZIs. The composite of organic molecules and nanocarriers is an optional strategy to cross the BBB and realize the imaging of special targets in the brain.

## Supporting Information Available

Additional experimental details, materials, methods, and results (PDF)
