# Supplementary material for: Benzothiazolium Derivative-Capped Silica Nanocomposites for β-Amyloid Imaging In Vivo
Source: Anal Chem. 2021 Sep 8;93(37):12617–27. doi: 10.1021/acs.analchem.1c02289 (PMC8746709; doi:10.1021/acs.analchem.1c02289)
Supplement: Supplementary file 1 — ac1c02289_si_001.pdf [file ac1c02289_si_001.pdf]

Supporting Information

Benzothiazolium Derivatives Capped Silica Nanocompites for β-amyloid Imaging *in vivo*

Lijun Ma, † Shu Yang, § Yufan Ma, † Yuzhi Chen, † Zhenguo Wang, † Tony D. James,*¶ Xuefei Wang,* ‡Zhuo Wang*†

† State Key Laboratory of Chemical Resource Engineering, College of Chemistry, Beijing Advanced Innovation Center for Soft Matter Science and Engineering, Beijing University of Chemical Technology, Beijing, 100029, China.

‡ School of Chemistry and Chemical Engineering, University of Chinese Academy of Sciences, Beijing, 100049, China.

¶ Department of Chemistry, University of Bath, BA2 7AY, UK.

§ Department of Pharmacy, Beijing Tiantan Hospital, Capital Medical University, Beijing, 100070, China.

†Corresponding author's fax: +86-01-64434898

†Corresponding emails: wangzhuo77@mail.buct.edu.cn

‡Corresponding emails:wangxf@ucas.ac.cn

¶Corresponding emails:T.D.James@bath.ac.uk

Table of Contents.

Table S1. Properties of four different MSN..S3
Table S2. The spectral data of probes...S3
Figure S1. The relationship between dihedral angel and transmission energy.............S4
Table S3. The relationship between oscillator strengthanddihedral angel...................S5
Table S4. The autodocking data between ThT and $A\beta_{1-42}$ aggregates model.............S5
Table S5. The autodocking data between SZI-1 and $A\beta_{1-42}$ aggregates model............S6
Table S6. The autodocking data between SZI-2 and $A\beta_{1-42}$ aggregates model............S6
Table S7. The autodocking data between SZI-3 and $A\beta_{1-42}$ aggregates model............S7
Figure S2. The viscosity response characteristics of SZIs..S7
Figure S3. The selectivity of SZIs...S7
Figure S4. TEMs of $A\beta_{1-42}$ aggregates...S8
Figure S5. The fluorescence response of ThT to $A\beta_{1-42}$ aggregates............................S8
Figure S6. *In vivo* BBB penetration test..S8
Table S8. Comparation of our probes with reported probes.......................................S9
Figure S7. The wide range TEM of $MSN-NH_2$..S10
Figure S8. The particle size distribution of different MSN nanoparticles...................S10
Figure S9. The FTIR image of before and after the removal of CTAC in MSN..........S10
Figure S10. The absorption spectrum of SZI-1 in different solvent...........................S12
Figure S11. The fluorescence intensity of SZI-1 in different solvent.........................S12
Figure S12. The absorption spectrum of SZI-2 in different solvent...........................S14
Figure S13. The fluorescence intensity of SZI-2 in different solvent.........................S14
Figure S14. The absorbance and fluorescence intensity of MSN-Lf@SZI-1..............S15
Figure S15. The absorbance and fluorescence intensity of MSN-Lf@SZI-2..............S15
Figure S16. The CLSM images of MSN-Lf@SZIs..S16
Figure S17. Photostability of SZI-1 and SZI-2..S16
Table S9. The conditions used for LC-MS analysis..S17
Table S10. Biodistribution of SZI-1 and SZI-2 in the brains of KM mice................S17
Figure S18.The representative LC-MS data of SZI-1..S18
Figure S19.The representative LC-MS data of SZI-2..S19
Figure S20. The representative mass spectrometry data of SZIs and SZIs in brain...S20
Figure S21-S32. Characterization data of probes...S21

Table S1. Properties of four different MSN.

Sample	TEA [g]	Pore diameter [nm]	TEM average diameter [nm]	DLS average diameter [nm]	PDI	Zeta potential [mV]
MSN	0.08	2.7	40	46.80	0.177	-20.08
MSN-NH_2	/	/	40	60.06	0.221	17.64
MSN-PEG	/	/	40	53.40	0.394	19.91
MSN-Lf	/	/	50	60.41	0.243	8.80
MSN-Lf@SZIs	/	/	50	/	/	5.67

Table S2. The spectral data of probes.

Probes	Sovents	Ex [nm]	Em [nm][a)]	Stokes shift [nm]	Absolute Quantum Yield [%]	FL Intensity (Em,with Aβ) [nm][a)]	Fold[b)]	Clog P[c)]	Kd [nM][d)]	ε [L·mol^{-1}cm^{-1}] [e)]
SZI-1	THF	527	600	73	19.2%	n.d.	3	0.20	507.6 ±94.75	6897.168
	Ethanol	526.5	592	65.5		n.d.				5926.568
	DMSO	524.5	608	83.5		n.d.				7038.538
	PDO	531	595	64		n.d.				8663.449
	PBS	510	593	83		2283				7481.385
SZI-2	THF	568.5	702	133.5	30.75%	n.d.	1.7	1.26	600.6 ±94.82	4944.128
	Ethanol	569.5	693	123.5		n.d.				4030.88
	DMSO	558.5	711	152.5		n.d.				5044.181
	PDO	575.5	692	116.5		n.d.				5574.688
	PBS	521	688	167		778.3				5058.816
SZI-3	THF	559	812	253	n.d.	n.d.	n.d.	2.19	n.d.	1872.063
	Ethanol	559	810	251		n.d.				3354.34
	DMSO	554	815	261		n.d.				3433.34
	PDO	561	809	248		n.d.				3986.34
	PBS	518	803	285		n.d.				3652.17

a) Set b = 10 for a specific emission wavelength.

b) The calculation of the fluorescence enhancement fold was calculated based on the fluorescence ratio before and after binding to Aβ.

c) The ClogP was calculated by ALOGPS 2.1 program

(http://146.107.217.178/lab/alogps/start.html)

n.d. refers to not determined.

d) Kd value refers to dissociation constant, which was given as best-fit values±Std. Error. The Kd value was calculated by Graphpad Prism 5.0 Software with nonlinear regression (one site-specific binding) method. The Kd was calculated by the following formula:

$$Y = \frac{B_{max} \cdot X}{k_d + X}$$

where X is the concentration of probes, Y is change in fluorescence intensity, B_{max} is the maximum specific binding has the same units as Y, Kd is the equilibrium binding constant.

e) ε was calculated by Lambert-Beer law.

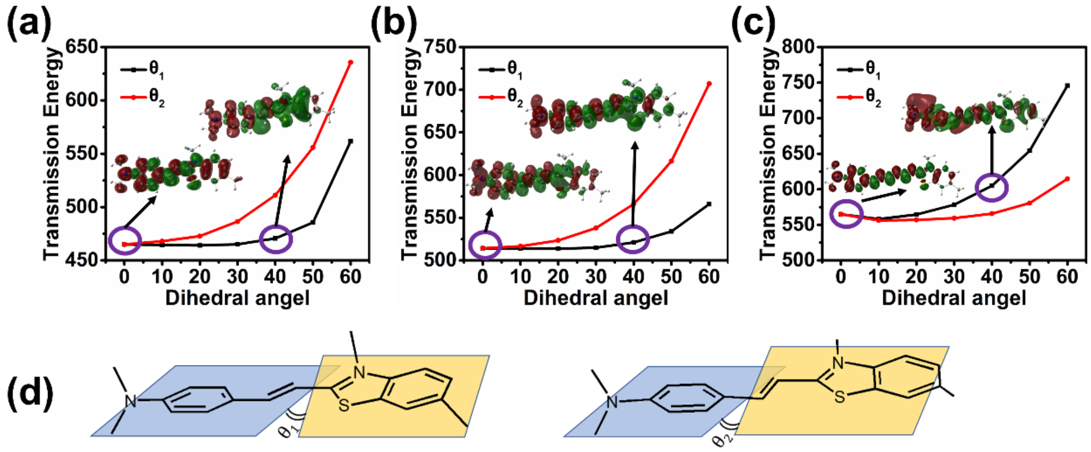

Figure S1. The relationship between dihedral angel and transmission energy. (a) SZI-1. (b) SZI-2. (c) SZI-3. (d) Description of the distortion method in the molecule used for calculation.

Table S3. The relationship between oscillator strength and dihedral angel.

Dihedral (°)	SZI-1		SZI-2		SZI-3	
	θ_1	θ_2	θ_1	θ_2	θ_1	θ_2
0	1.49	1.49	2.0	2.0	2.47	2.47
10	1.5	1.46	2.0	1.97	2.53	2.57
20	1.46	1.42	1.96	1.89	2.49	2.4
30	1.4	1.3	1.8	1.71	2.4	2.2
40	1.27	1.1	1.7	1.44	2.19	1.8
50	1.0	0.8	1.4	1.08	1.89	1.36
60	0.6	0.5	0.9	0.7	1.31	0.87

Table S4. The autodocking data between ThT and Aβ_{1-42} aggregates model.

Rank SunRank	Binding Energy [kcal/mol]	Torsional Energy [kcal/mol]	Intermolecular Energy [kcal/mol]	Internal Energy [kcal/mol]	Unbound Extended Energy [kcal/mol]
1_1	-6.63	0.6	-7.23	-0.39	-0.39
1_2	-6.6	0.6	-7.2	-0.42	-0.42
1_3	-6.51	0.6	-7.11	-0.38	-0.38
1_4	-6.51	0.6	-7.1	-0.41	-0.41
2_1	-6.19	0.6	-6.79	-0.51	-0.51
3_1	-5.71	0.6	-6.3	-0.45	-0.45
4_1	-5.68	0.6	-6.27	-0.41	-0.41
5_1	-5.63	0.6	-6.22	-0.38	-0.38
6_1	-5.29	0.6	-5.88	-0.52	-0.52
7_1	-5.23	0.6	-5.83	-0.52	-0.52

Table S5. The autodocking data between SZI-1 and Aβ_{1-42} aggregates model.

Rank SunRank	Binding Energy [kcal/mol]	Torsional Energy [kcal/mol]	Intermolecular Energy [kcal/mol]	Internal Energy [kcal/mol]	Unbound Extended Energy [kcal/mol]
1_1	-6.72	0.89	-7.61	-0.48	-0.48
1_2	-6.57	0.89	-7.47	-0.47	-0.47
1_3	-6.57	0.89	-7.47	-0.48	-0.48
1_4	-6.56	0.89	-7.45	-0.49	-0.49
1_5	-6.54	0.89	-7.44	-0.49	-0.49
1_6	-6.43	0.89	-7.32	-0.47	-0.47
1_7	-6.34	0.89	-7.24	-0.48	-0.48
1_8	-6.32	0.89	-7.21	-0.28	-0.28
1_9	-6.28	0.89	-7.18	-0.48	-0.48
1_10	-6.04	0.89	-6.94	-0.48	-0.48

Table S6. The autodocking data between SZI-2 and Aβ_{1-42} aggregates model.

Rank SunRank	Binding Energy [kcal/mol]	Torsional Energy [kcal/mol]	Intermolecular Energy [kcal/mol]	Internal Energy [kcal/mol]	Unbound Extended Energy [kcal/mol]
1_1	-7.37	1.19	-8.56	-0.61	-0.61
1_2	-7.31	1.19	-8.5	-0.6	-0.6
1_3	-7.17	1.19	-8.36	-0.51	-0.51
1_4	-7.1	1.19	-8.29	-0.61	-0.61
1_5	-7.09	1.19	-8.29	-0.61	-0.61
1_6	-7.05	1.19	-8.25	-0.55	-0.55
1_7	-7.04	1.19	-8.23	-0.55	-0.55
1_8	-6.98	1.19	-8.17	-0.55	-0.55
1_9	-6.98	1.19	-8.17	-0.62	-0.62
1_10	-6.94	1.19	-8.14	-0.62	-0.62

Table S7. The autodocking data between SZI-3 and $A\beta_{1-42}$ aggregates model.

Rank SunRank	Binding Energy [kcal/mol]	Torsional Energy [kcal/mol]	Intermolecular Energy [kcal/mol]	Internal Energy [kcal/mol]	Unbound Extended Energy [kcal/mol]
1_1	-8.27	1.49	-9.77	-0.8	-0.8
1_2	-8.12	1.49	-9.61	-0.81	-0.81
1_3	-8.03	1.49	-9.52	-0.81	-0.81
2_1	-8.04	1.49	-9.53	-0.78	-0.78
3_1	-8.04	1.49	-9.53	-0.8	-0.8
4_1	-7.87	1.49	-9.36	-0.79	-0.79
5_1	-7.56	1.49	-9.06	-0.79	-0.79
6_1	-7.46	1.49	-8.95	-0.8	-0.8
7_1	-6.86	1.49	-8.35	-0.82	-0.82
8_1	-6.73	1.49	-8.22	-0.81	-0.81

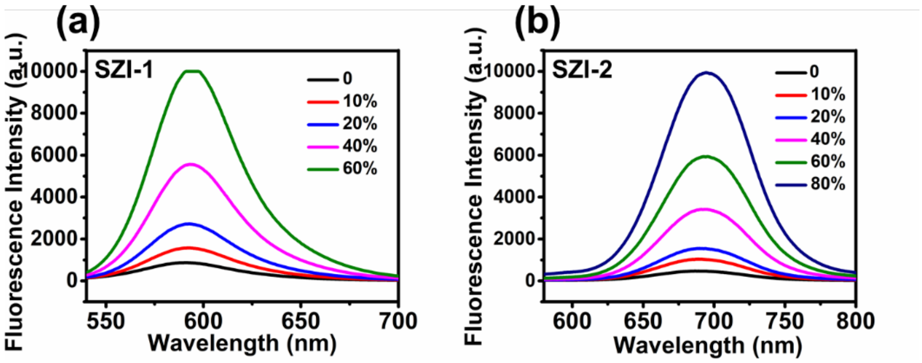

Figure S2. The viscosity response characteristics of SZIs. (a) SZI-1. (b) SZI-2. The solvent was different ratios of 1,2-propanediol and water (v/v).

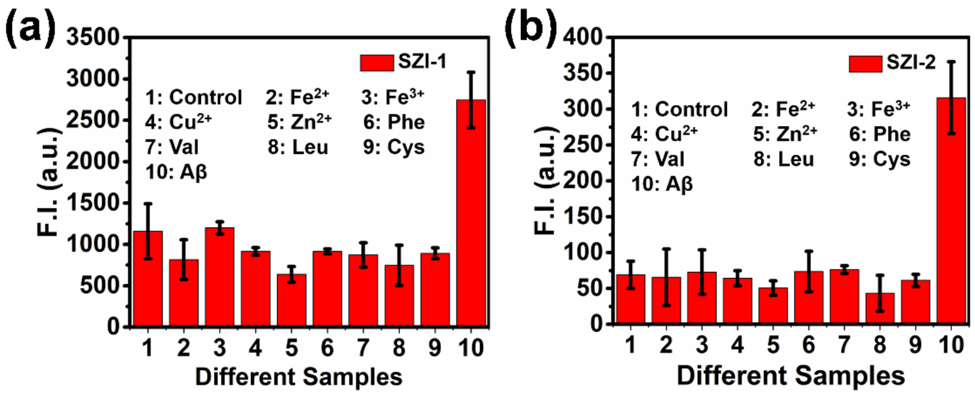

Figure S3. The selectivity of (a) SZI-1 (0.2 μg), (b) SZI-2 (0.2 μg) to different ions (20 μM), amino acid (20 μM) and $A\beta_{1-42}$ aggregates (20 μM). All the samples were tested in triplicate.

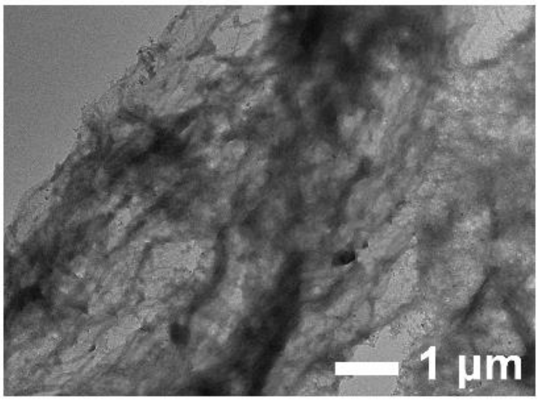

Figure S4. TEMs of $A\beta_{1-42}$ aggregates.

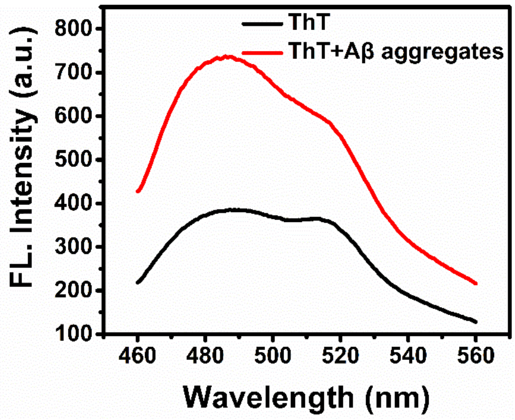

Figure S5. The fluorescence response of ThT to $A\beta_{1-42}$ aggregates.

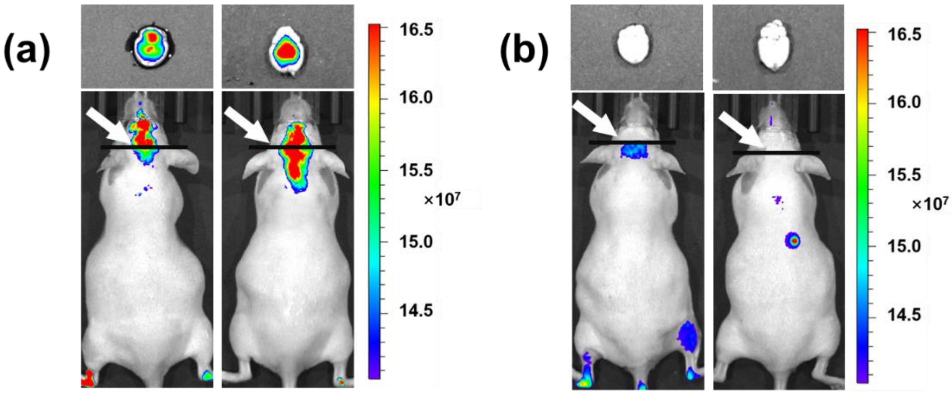

Figure S6. *In vivo* BBB penetration test. (a) The BBB penetration test using MSN-Lf@SZI-1 (left, 2 mg/kg in 50 % 1,2-propanediol and 50 % PBS) and MSN-Lf@SZI-2 (right, 2 mg/kg in 50 % 1,2-propanediol and 50 % PBS) in KM mice. (b) The BBB penetration test using SZI-1 (left, 2 mg/kg in 50 % 1,2-propanediol and 50 % PBS) and SZI-2 (right, 2 mg/kg in 50 % 1,2-propanediol and 50 % PBS) in KM mice. To be more intuitive, we also performed fluorescence imaging on the dissected mouse brain (the small images above in a and b)

Table S8. Comparation of our probes with reported probes.

	Probe	Molecule structure	Kd	Target Analyte	Reference
1	ANCA	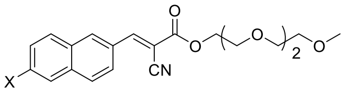	1.4±0.2 ~ 13.8±3.1 μM	Aβ fibrils	(1)
2	ThT	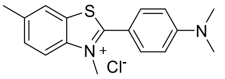	890 nM	Aβ fibrils	(2) (3)
3	NMM	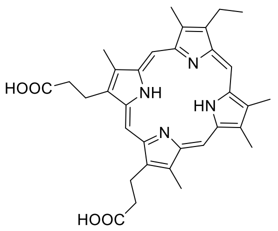	>2 μM	Aβ fibrils	(4)
4	AN-SP	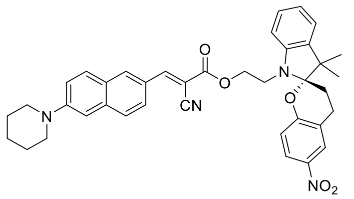	1.7 μM	Aβ oligomer	(5)
5	F-SLOH	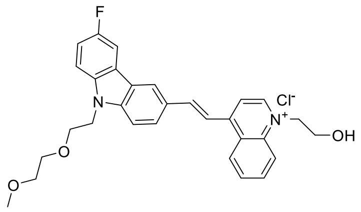	1.90 μM<br>0.66 μM	Aβ fibrils<br>Aβ oligomer	(6)
6	ARCAM 1	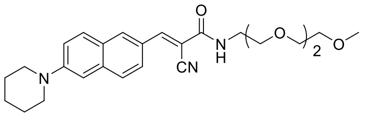	870±280 nM	Aβ fibrils	(7)
7	4c<br>4d	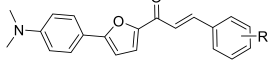 4c, R=4-B(OH)2<br>4d, R=3-B(OH)2	4c, 0.79 ± 0.05 μM<br>4d, 0.90 ± 0.02 μM	Aβ fibrils	(8)
8	SZI-1	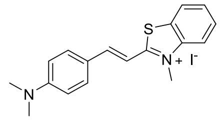	507.6±94.75 nM	Aβ fibrils	This work
9	SZI-2	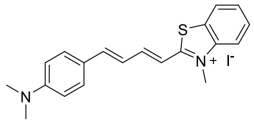	600.6±94.82 nM	Aβ fibrils	This work

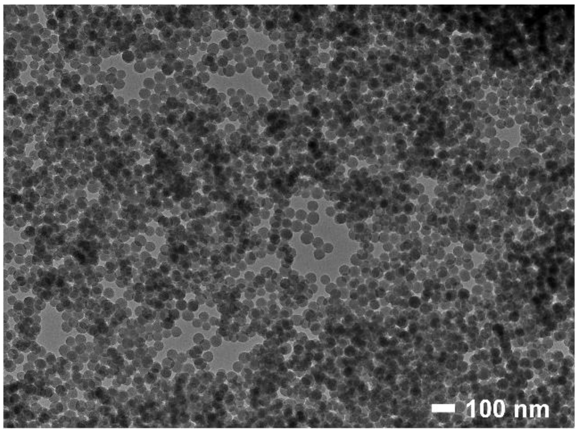

Figure S7. The wide range TEM of MSN-NH_2. Scale bar is 100 nm.

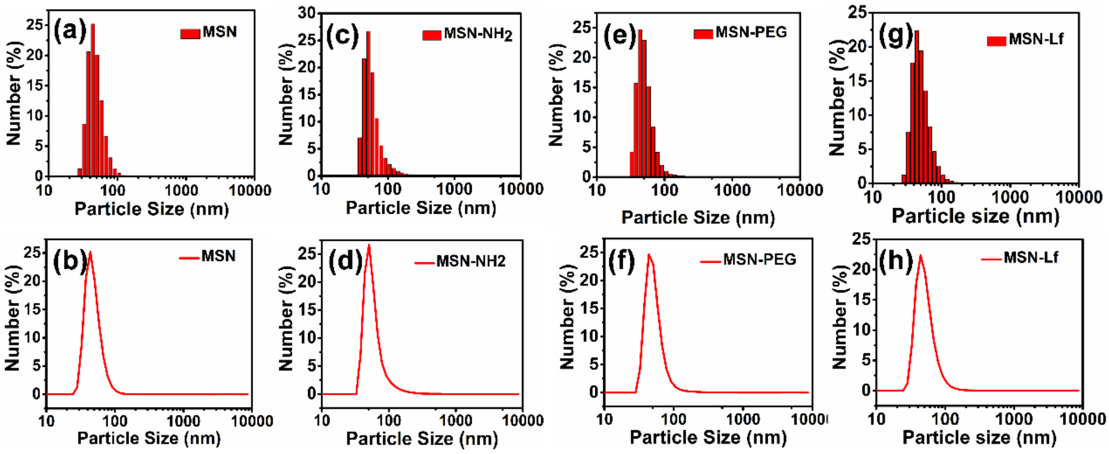

Figure S8. The particle size distribution of different MSN nanoparticles. (a, b) The particle size distribution of MSN. (c, d) The particle size distribution of MSN-NH_2. (e, f) The particle size distribution of MSN-PEG. (g, h) The particle size distribution of MSN-Lf.

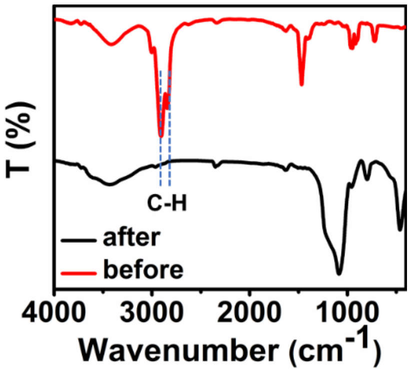

Figure S9. The FTIR image of before and after the removal of CTAC in MSN.

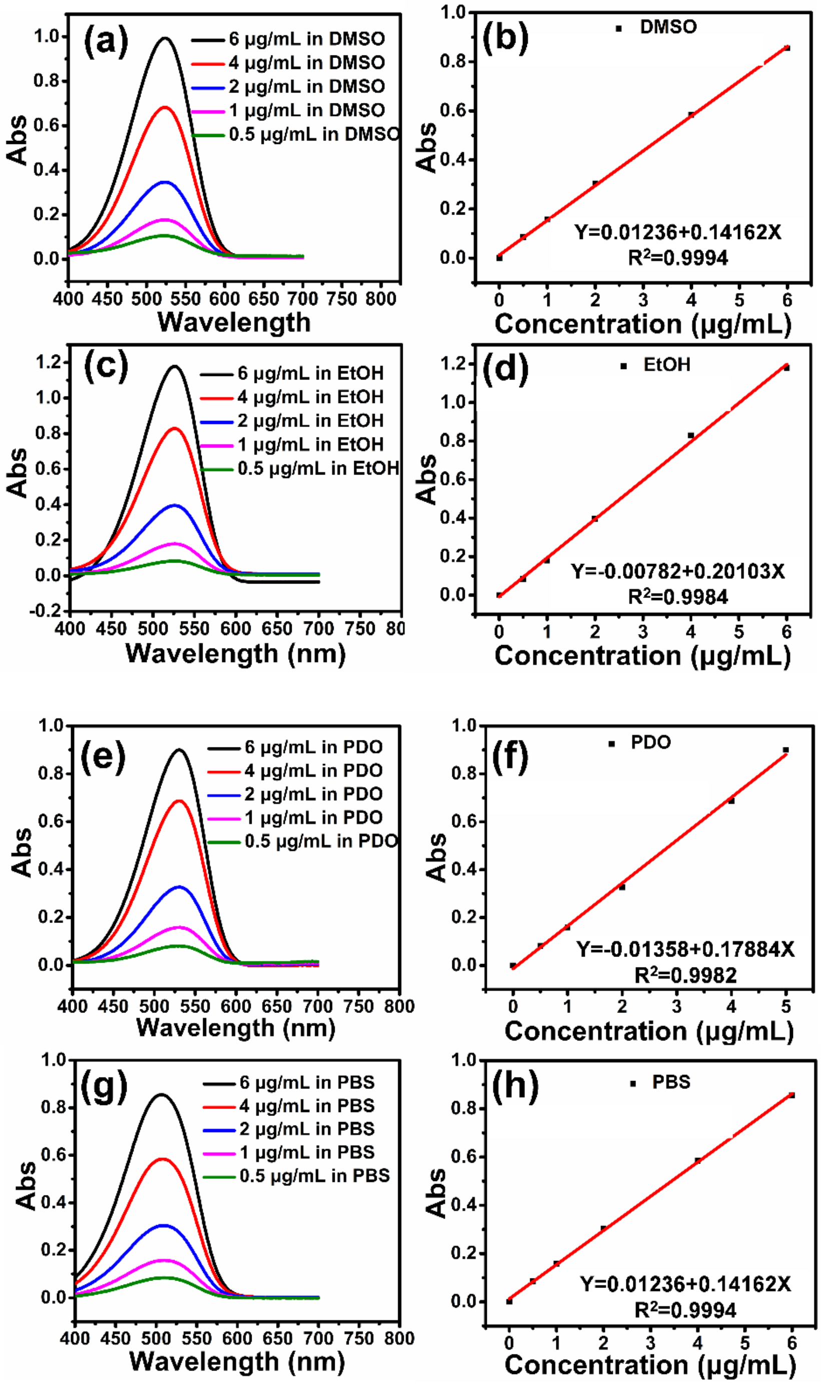

(a)
6 μg/mL in DMSO
4 μg/mL in DMSO
2 μg/mL in DMSO
1 μg/mL in DMSO
0.5 μg/mL in DMSO
Abs
Wavelength
(b)
DMSO
Y=0.01236+0.14162X
R²=0.9994
Concentration (μg/mL)
(c)
6 μg/mL in EtOH
4 μg/mL in EtOH
2 μg/mL in EtOH
1 μg/mL in EtOH
0.5 μg/mL in EtOH
Wavelength (nm)
(d)
EtOH
Y=-0.00782+0.20103X
R²=0.9984
(e)
6 μg/mL in PDO
4 μg/mL in PDO
2 μg/mL in PDO
1 μg/mL in PDO
0.5 μg/mL in PDO
(f)
PDO
Y=-0.01358+0.17884X
R²=0.9982
(g)
6 μg/mL in PBS
4 μg/mL in PBS
2 μg/mL in PBS
1 μg/mL in PBS
0.5 μg/mL in PBS
(h)
PBS
Y=0.01236+0.14162X
R²=0.9994

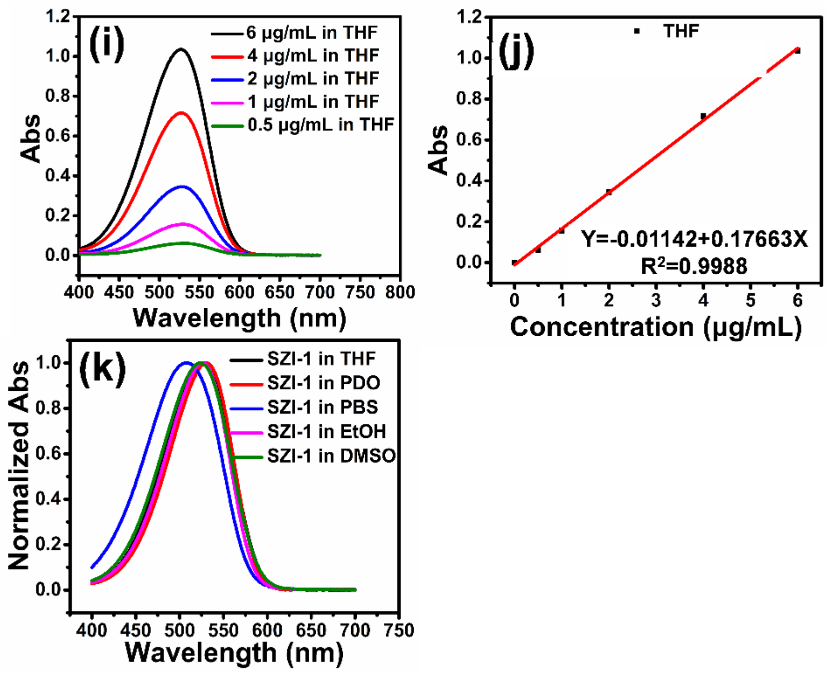

Figure S10. The absorption spectrum and corresponding standard curves of SZI-1 in different solvent. (a, b) DMSO, (c, d) EtOH, (e, f) PDO, (g, h) PBS, (i, j) THF. (k) Normalized Abs of SZI-1 in different solvent. The concentration has been given in the figure.

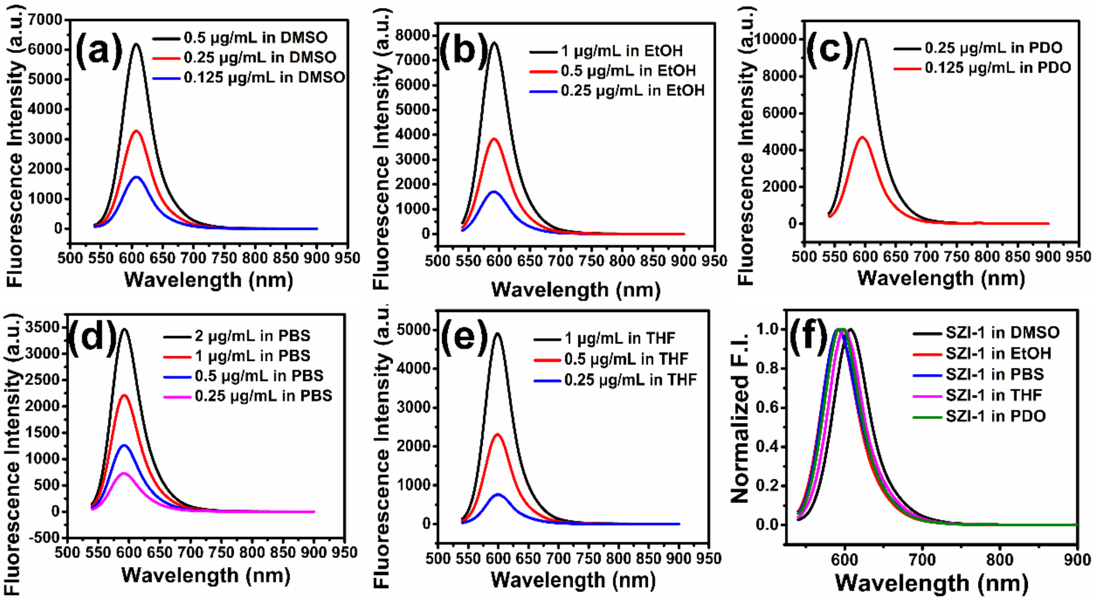

Figure S11. The fluorescence intensity of SZI-1 in different solvent. (a) DMSO, (b) EtOH, (c) PDO, (d)PBS (e) THF. (f) Normalized FL of SZI-1 in different solvent. The concentration has been given in the figure.

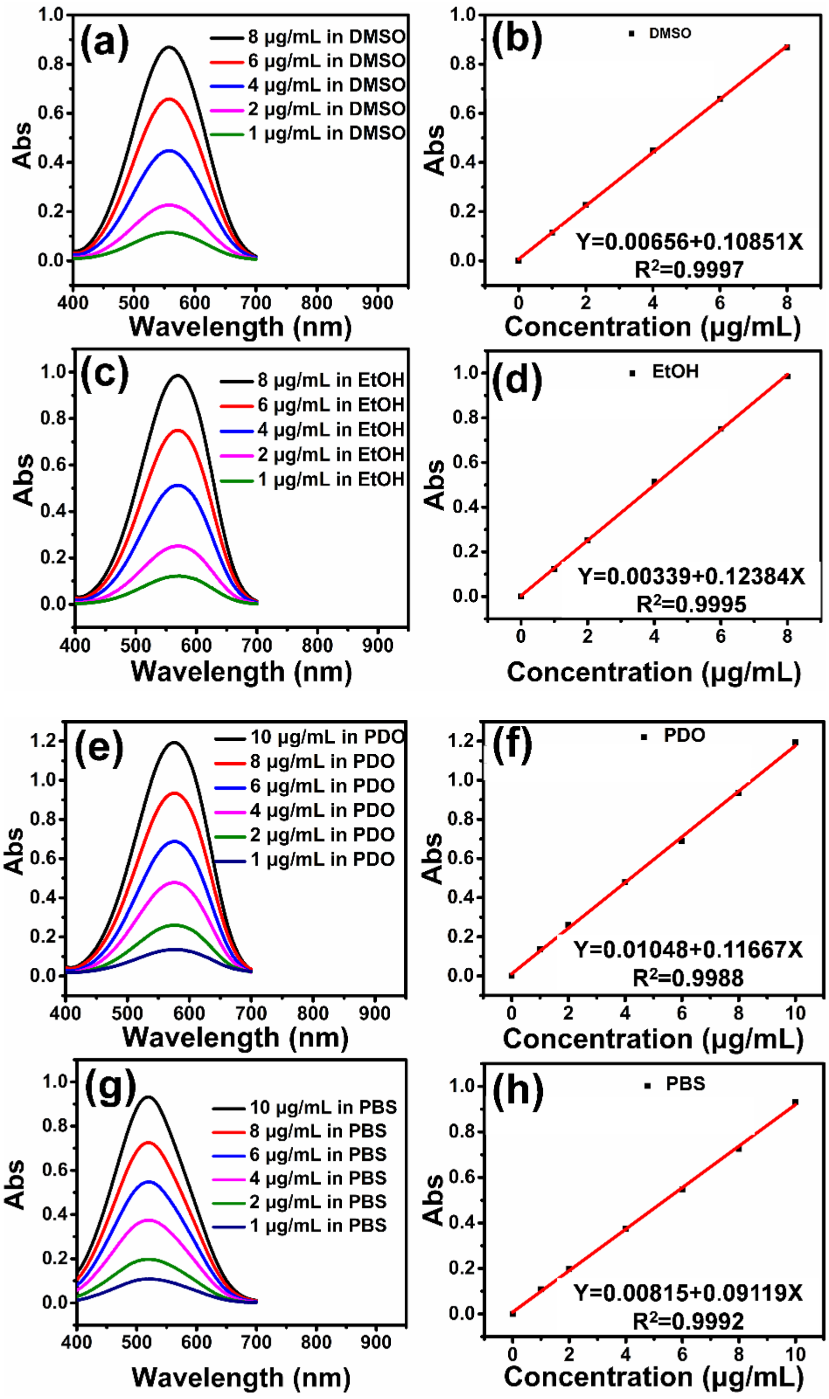
(a)
8 μg/mL in DMSO
6 μg/mL in DMSO
4 μg/mL in DMSO
2 μg/mL in DMSO
1 μg/mL in DMSO
Abs
Wavelength (nm)
(b)
DMSO
Y=0.00656+0.10851X
R²=0.9997
Concentration (μg/mL)
(c)
8 μg/mL in EtOH
6 μg/mL in EtOH
4 μg/mL in EtOH
2 μg/mL in EtOH
1 μg/mL in EtOH
(d)
EtOH
Y=0.00339+0.12384X
R²=0.9995
(e)
10 μg/mL in PDO
8 μg/mL in PDO
6 μg/mL in PDO
4 μg/mL in PDO
2 μg/mL in PDO
1 μg/mL in PDO
(f)
PDO
Y=0.01048+0.11667X
R²=0.9988
(g)
10 μg/mL in PBS
8 μg/mL in PBS
6 μg/mL in PBS
4 μg/mL in PBS
2 μg/mL in PBS
1 μg/mL in PBS
(h)
PBS
Y=0.00815+0.09119X
R²=0.9992

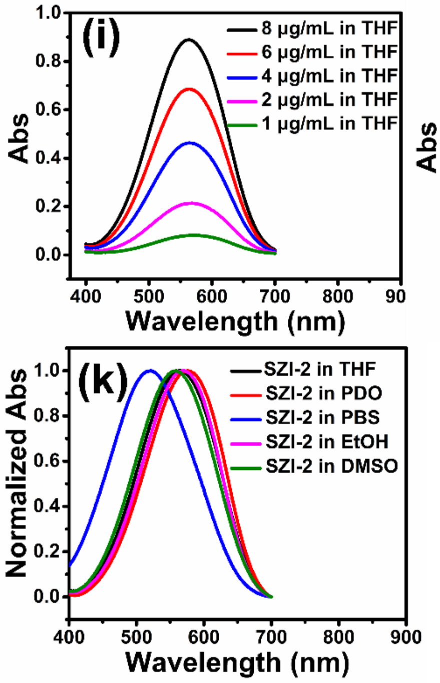

Figure S12. The absorption spectrum and corresponding standard curves of SZI-2 in different solvent. (a, b) DMSO, (c, d) EtOH, (e, f) PDO, (g, h) PBS, (i, j) THF. (k) Normalized Abs of SZI-2 in different solvent. The concentration has been given in the figure.

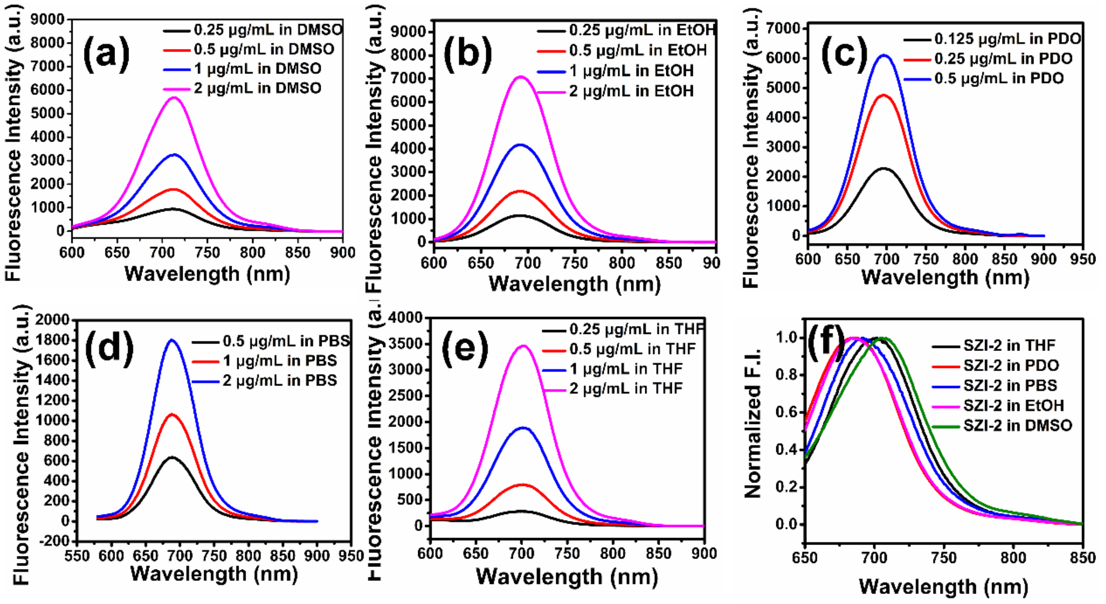

Figure S13. The fluorescence intensity of SZI-2 in different solvent. (a) DMSO, (b) EtOH, (c) PDO, (d) PBS, (e) THF. (f) Normalized FL of SZI-2 in different solvent. The concentration has been given in the figure.

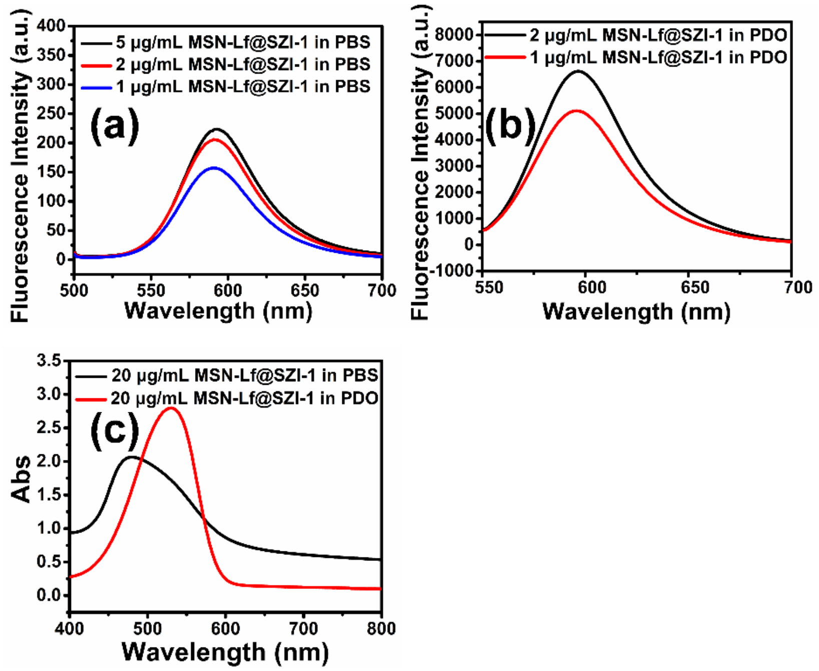

Figure S14. The fluorescence intensity of MSN-Lf@SZI-1 in (a) PBS and (b) PDO. (c) The Abs of MSN-Lf@SZI-1. The concentration has been given in the figure.

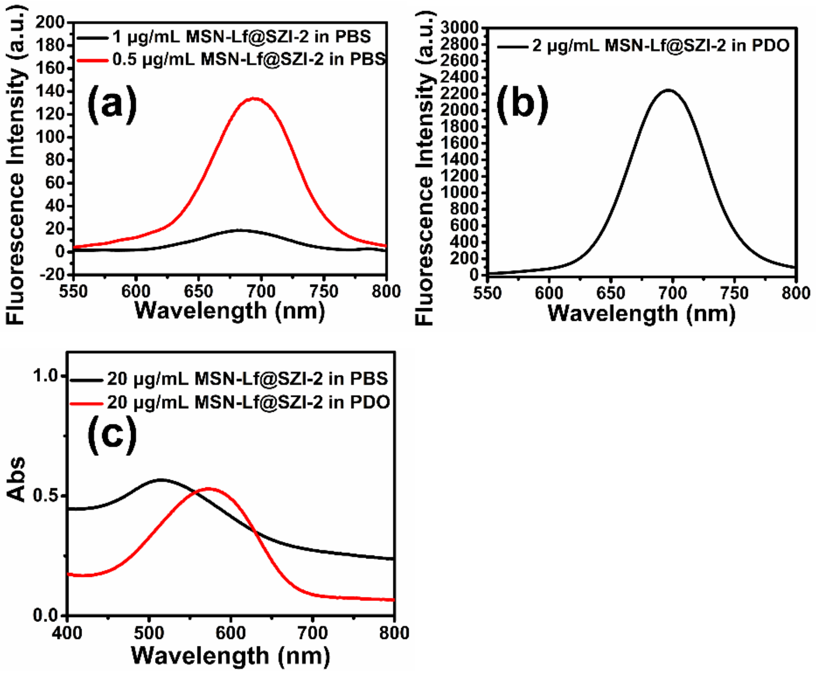

Figure S15. The fluorescence intensity of MSN-Lf@SZI-2 in (a) PBS and (b) PDO. (c) The Abs of MSN-Lf@SZI-2. The concentration has been given in the figure.

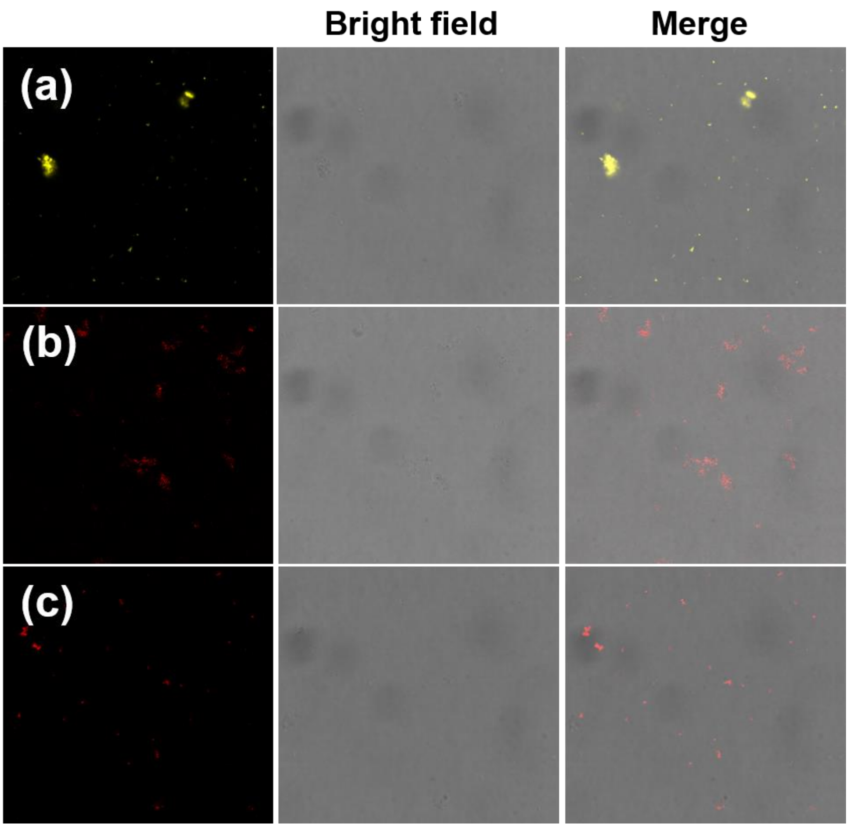

Figure S16. The confocal laser scanning microscope (CLSM) images of (a) MSN-Lf@SZI-1 (b) MSN-Lf@SZI-2 (c) MSN-Lf@SZI-3. The excitation wavelength (λ_{ex}) were 488 nm (a), 552 nm (b, c), respectively.

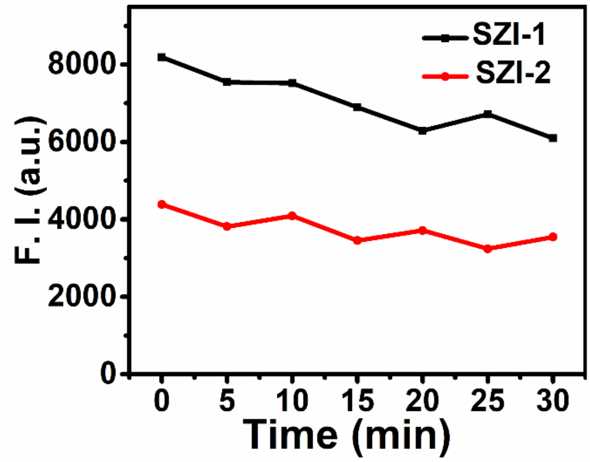

Figure S17. Photostability of SZI-1 and SZI-2 under continuous white light (15-16 mV) illumination.

Table S9. The conditions used for LC-MS analysis.

Probe	LC-MS analysis			
	Eluent CH_3CN: H_2O	Retention Time [min]	Analysis Time [min]	UV detector [nm]
SZI-1	5:95 ~ 95:5	12.165	32	254
SZI-1 in brain	5:95 ~ 95:5	12.635	32	254
SZI-2	5:95 ~ 95:5	13.128	32	254
SZI-2 in brain	5:95 ~ 95:5	12.967	32	254

Table S10. Uptake of SZI-1 and SZI-2 in the brains of KM mice.

Probe	Concentration [μg / mL] [a]	Intravenous Injection Volume [μL]	LC-MS injection volume [μL]	Peak Area [c]	The quality of the probe in the brain [μg]	Wet weight of brain [g]	Uptake [% ID/g]	Mean Uptake [% ID/g] [b]
SZI-1	47.5	125	50	30.82	0.64	0.42	25.47	15.73 ± 8.51
SZI-1	47.5	125	50	20.66	0.30	0.42	11.99	
SZI-1	47.5	125	50	18.96	0.24	0.42	9.73	
SZI-2	40	125	50	21.86	0.19	0.42	7.47	7.09 ± 0.79
SZI-2	40	125	50	8.31	0.13	0.42	6.19	
SZI-2	40	125	50	15.12	0.16	0.42	7.62	

a) The concentration actually refers to the concentration of the probe in MSN-Lf@SZIs, which was qualified by a UV-visible spectrophotometer according to standard curve.

b) % ID/g means % injection dose per gram of brain wet weight.

c) The peak area was determined by Origin 8.0 sofware.

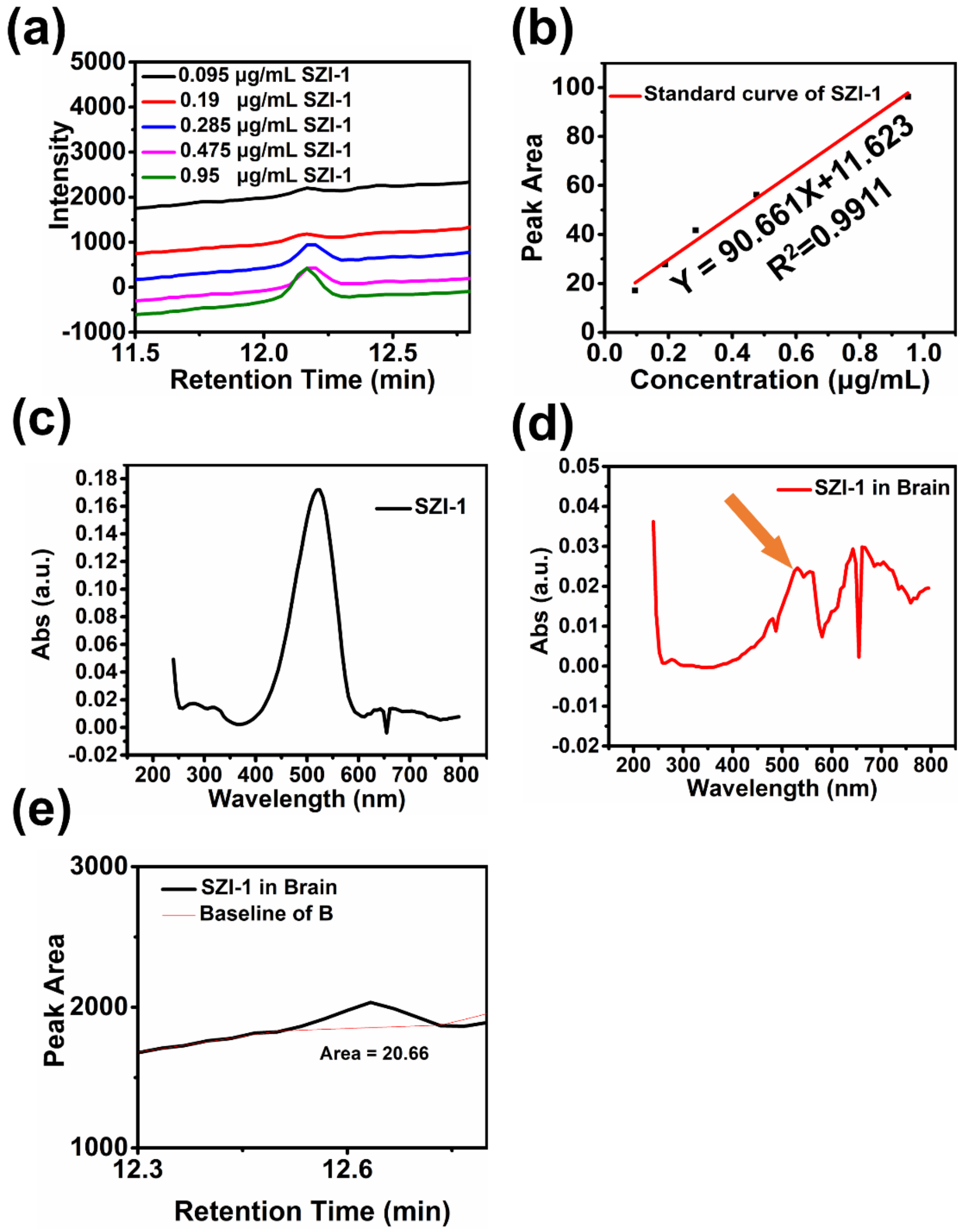

Figure S18.The representative LC-MS data of SZI-1. (a) The LC-MS chromatogram curve of SZI-1. (b) The standard curve of SZI-1. (c) The UV-Vis spectrometry of SZI-1 detected by LC-MS. (d) The UV-Vis spectrometry of SZI-1 in brain detected by LC-MS. (e) The representative peak area of SZI-1 in brain. The retention time was determined by mass spectrometry data of SZI-1 detected by LC-MS. The mass spectrometry data (ESI-MS) was given in Figure S20.

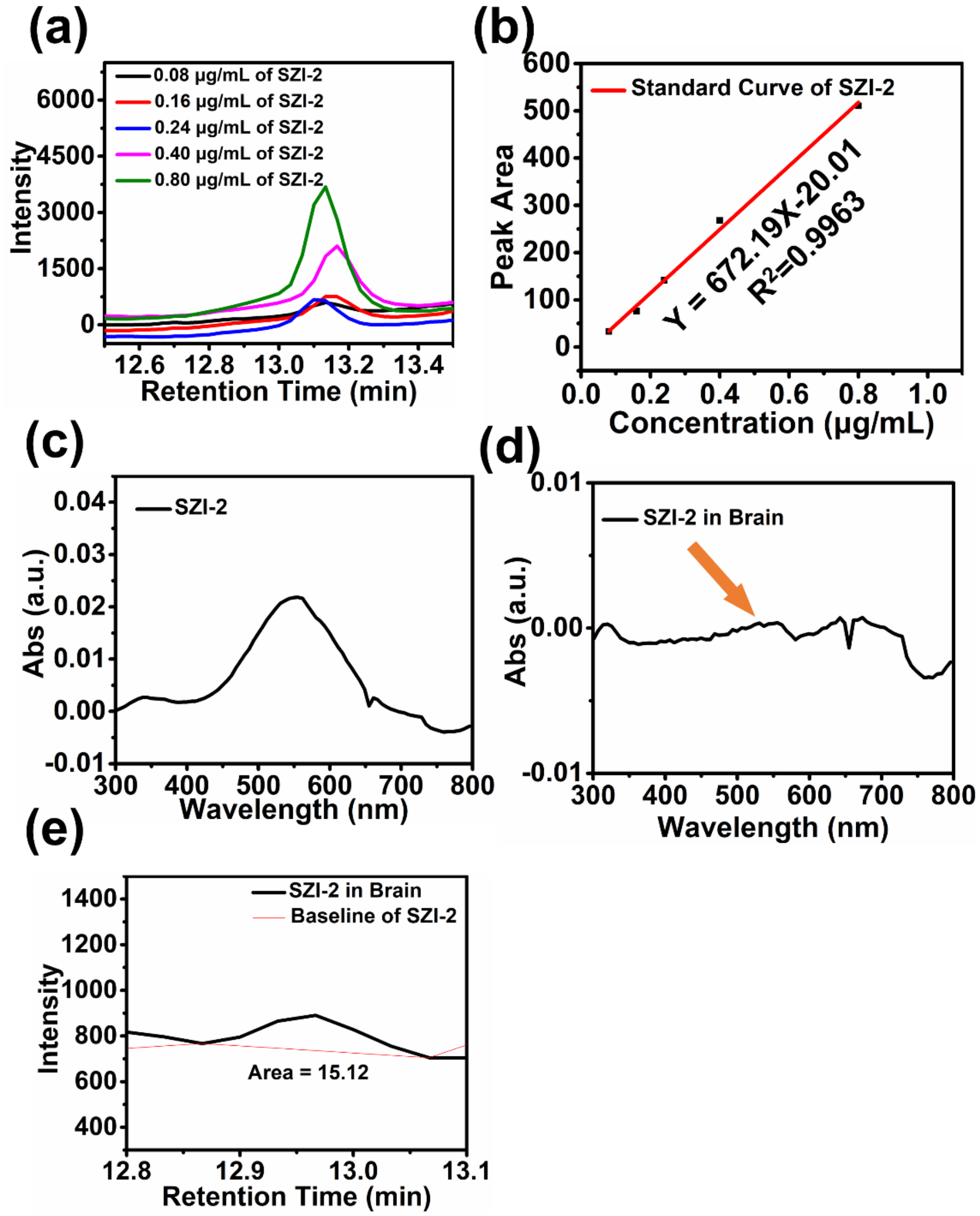

Figure S19. The representative LC-MS data of SZI-2. (a) The LC-MS chromatogram curve of SZI-2. (b) The standard curve of SZI-2. (c) The UV-Vis spectrometry of SZI-2 detected by LC-MS. (d) The UV-Vis spectrometry of SZI-2 in brain detected by LC-MS. (e) The representative peak area of SZI-2 in brain. The retention time was determined by mass spectrometry data of SZI-2 detected by LC-MS. The mass spectrometry data (ESI-MS) was given in Figure S20.

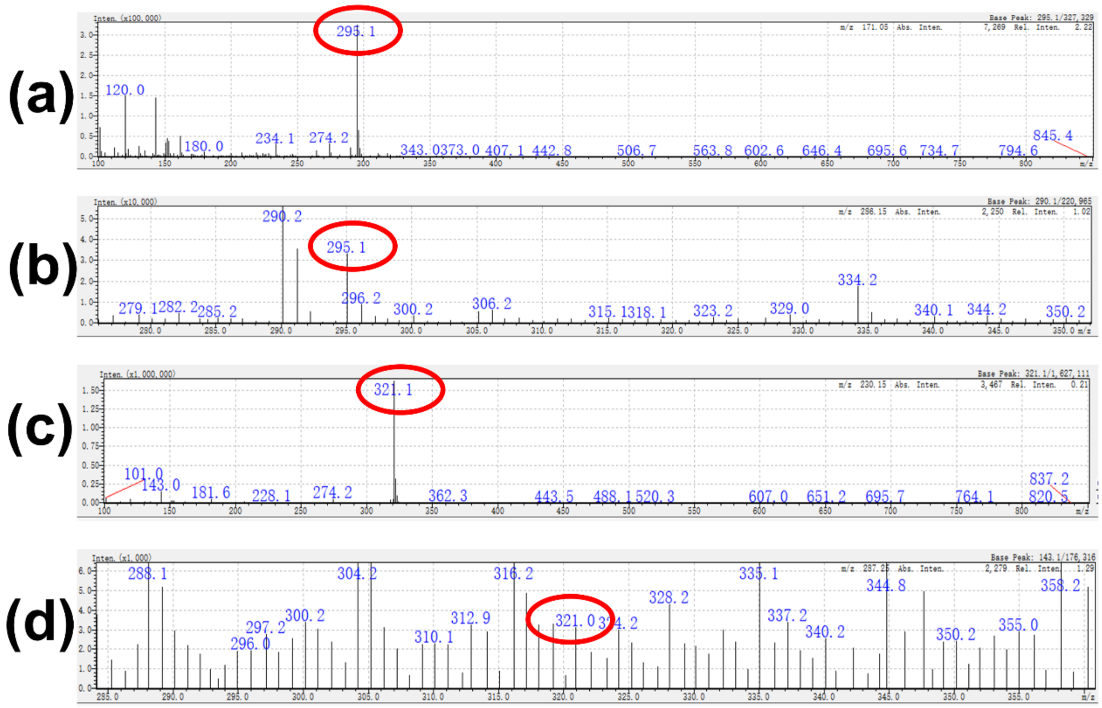

Figure S20. The representative mass spectrometry data of SZIs and SZIs in brain. (a) The mass spectrometry data of SZI-1. (b) The mass spectrometry data of SZI-1 in brain. (c) The mass spectrometry data of SZI-2. (d) The mass spectrometry data of SZI-2 in brain.

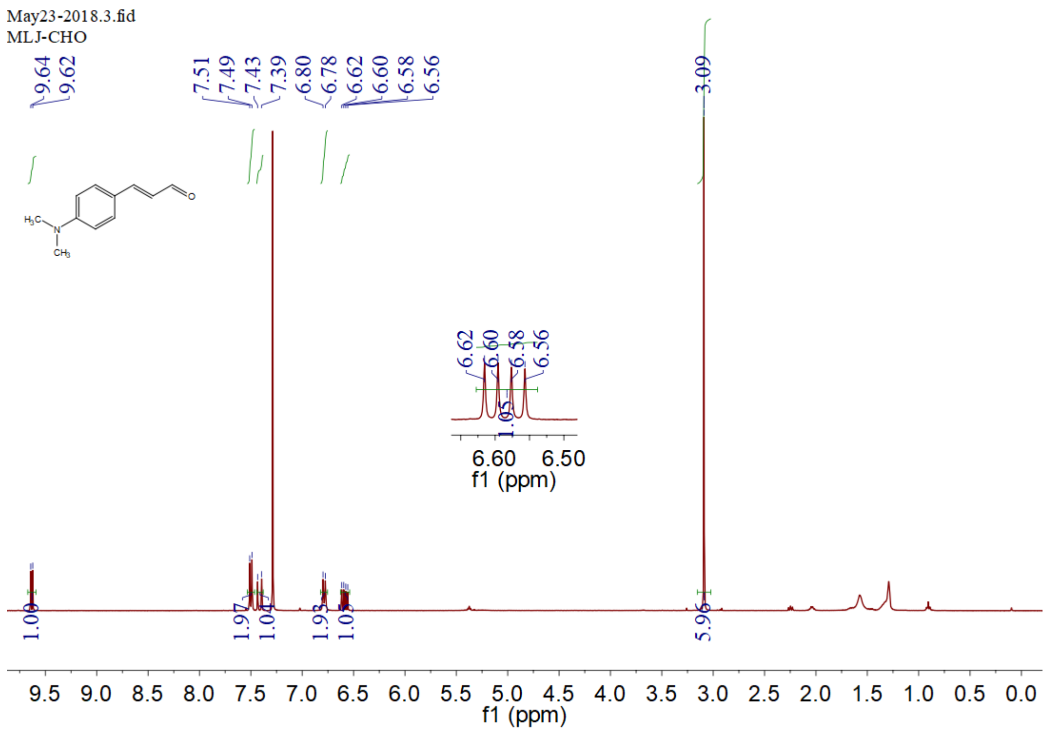

Figure S21. The ^{1}H NMR of compound 1.

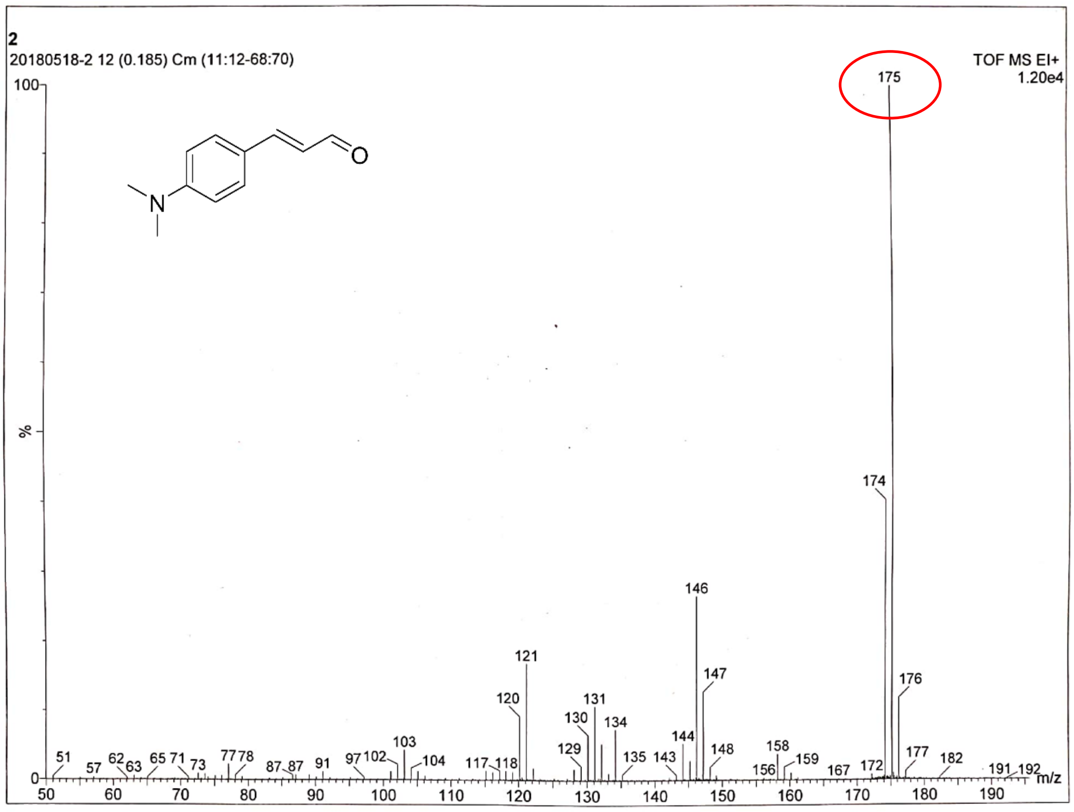

Figure S22. The mass Spectra (EI) of compound 1.

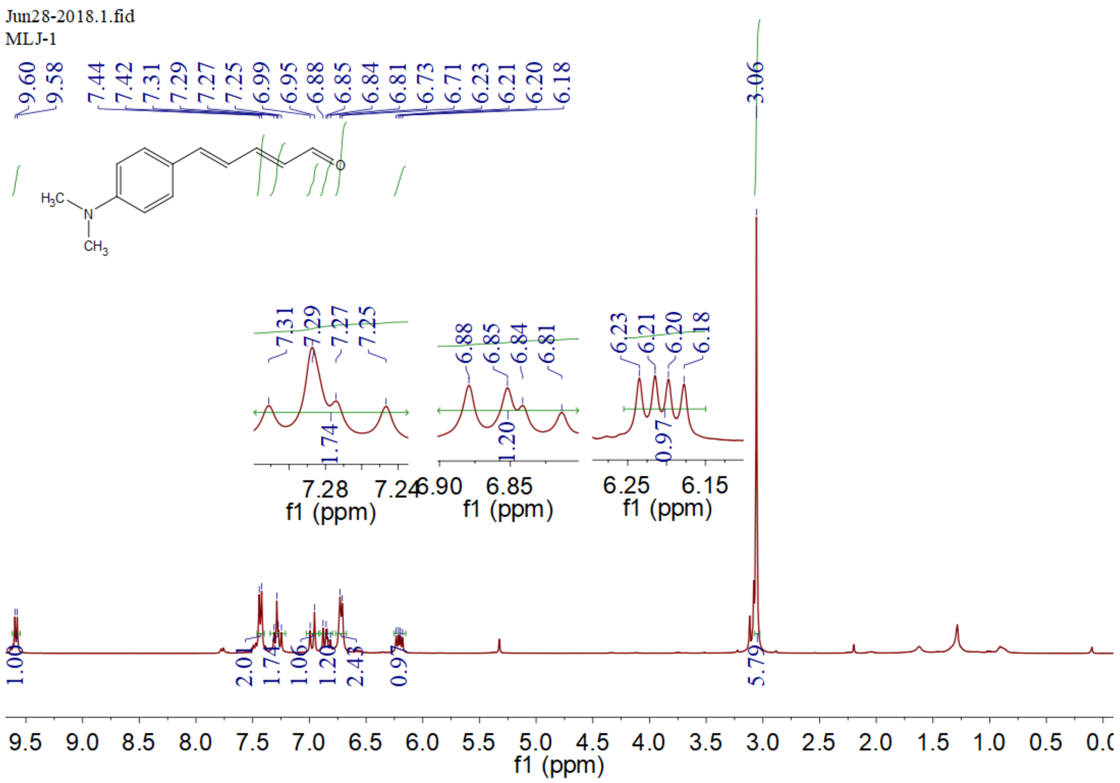

Figure S23. The ^{1}H NMR of compound 2.

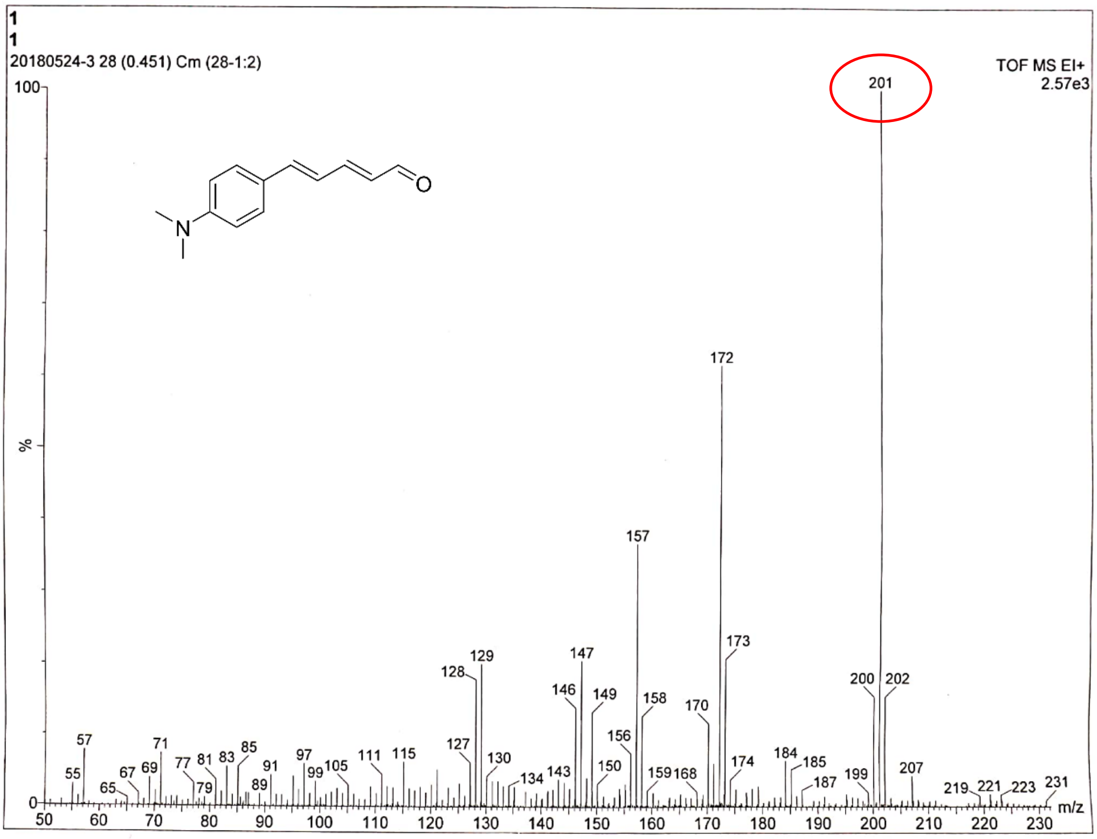

Figure S24. The mass Spectra (EI) of compound 2.

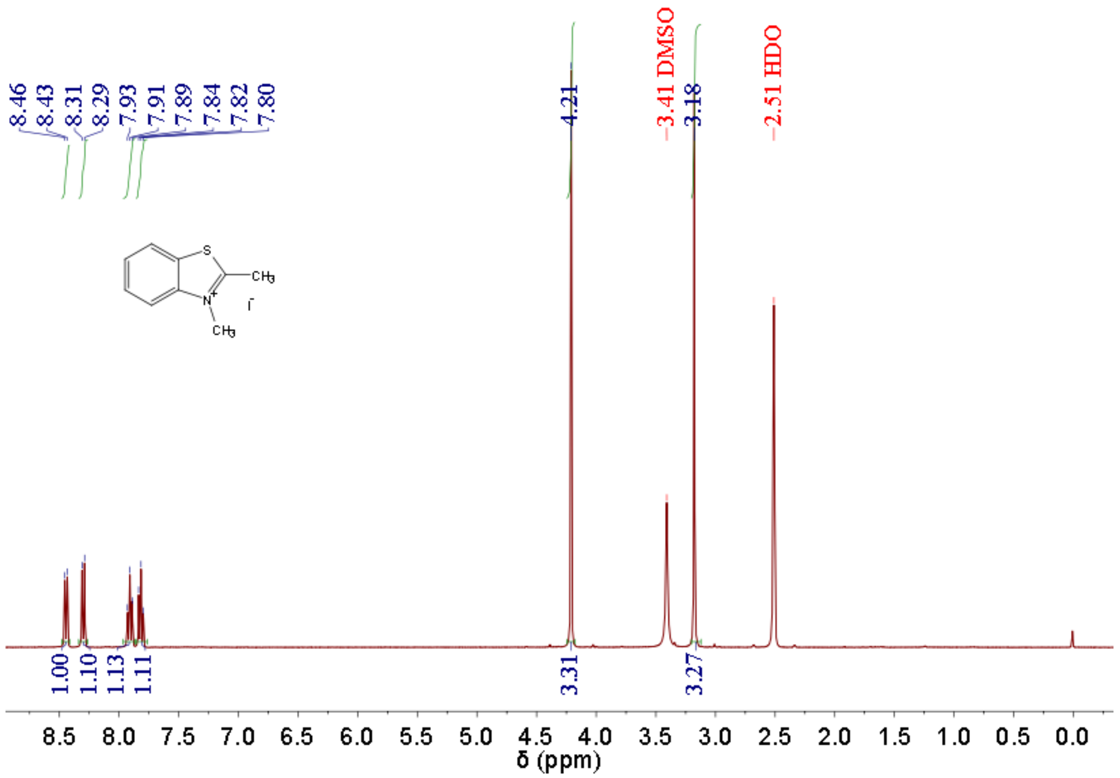

Figure S25. The ^{1}H NMR of compound 3.

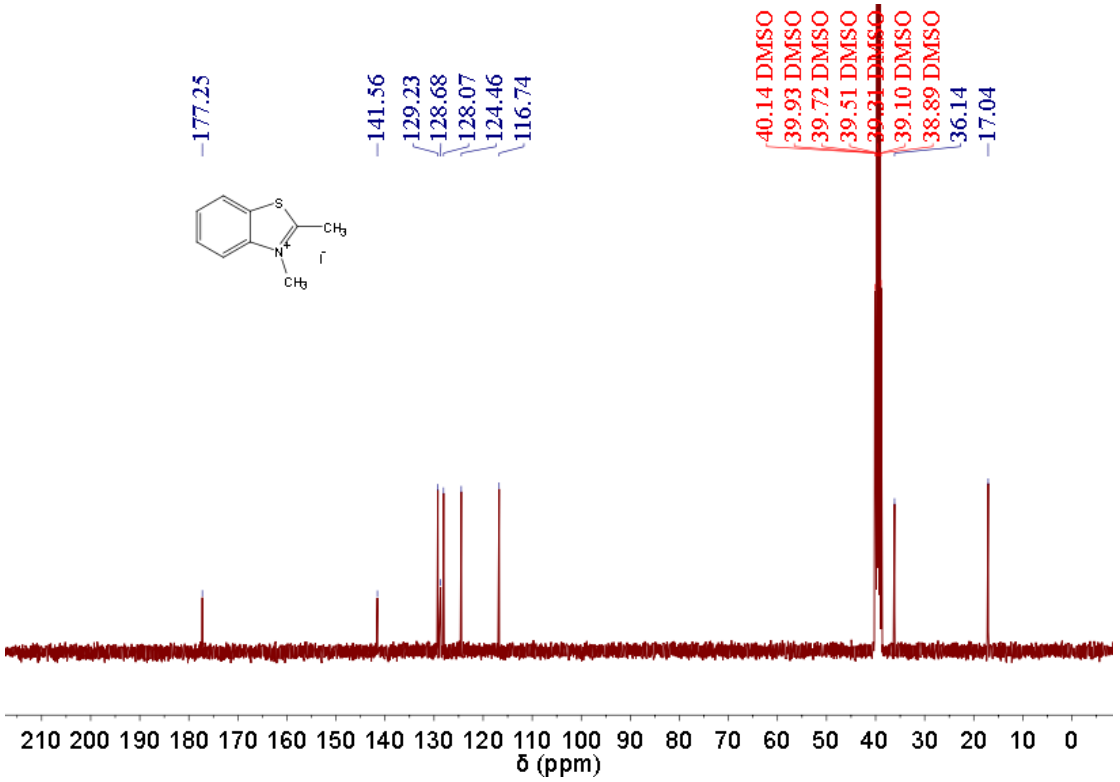

Figure S26. The ^{13}C NMR of compound 3.

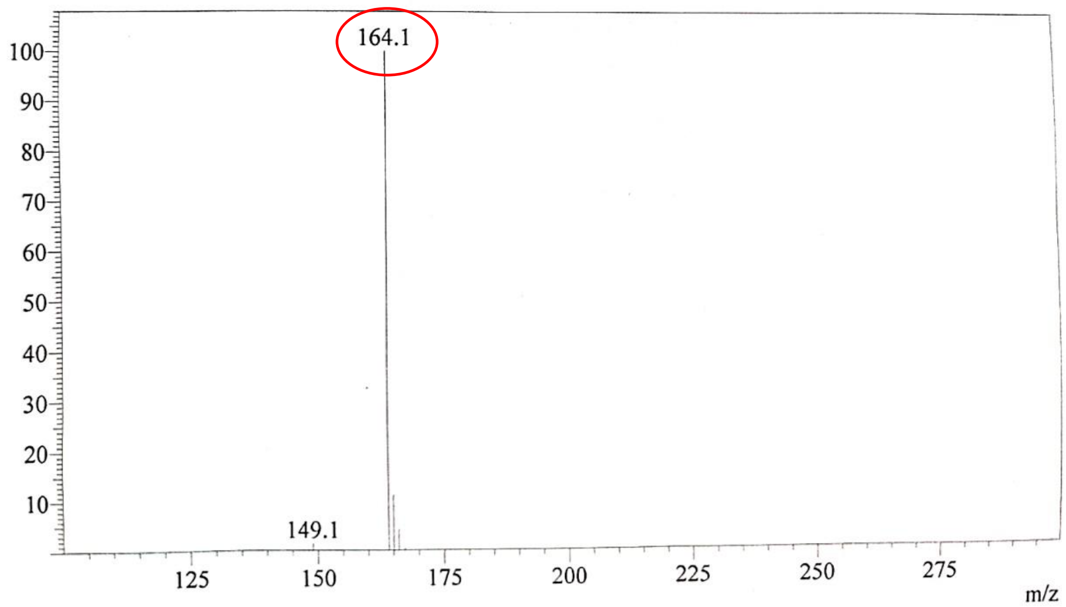

Figure S27. The mass Spectra (EI) of compound 3.

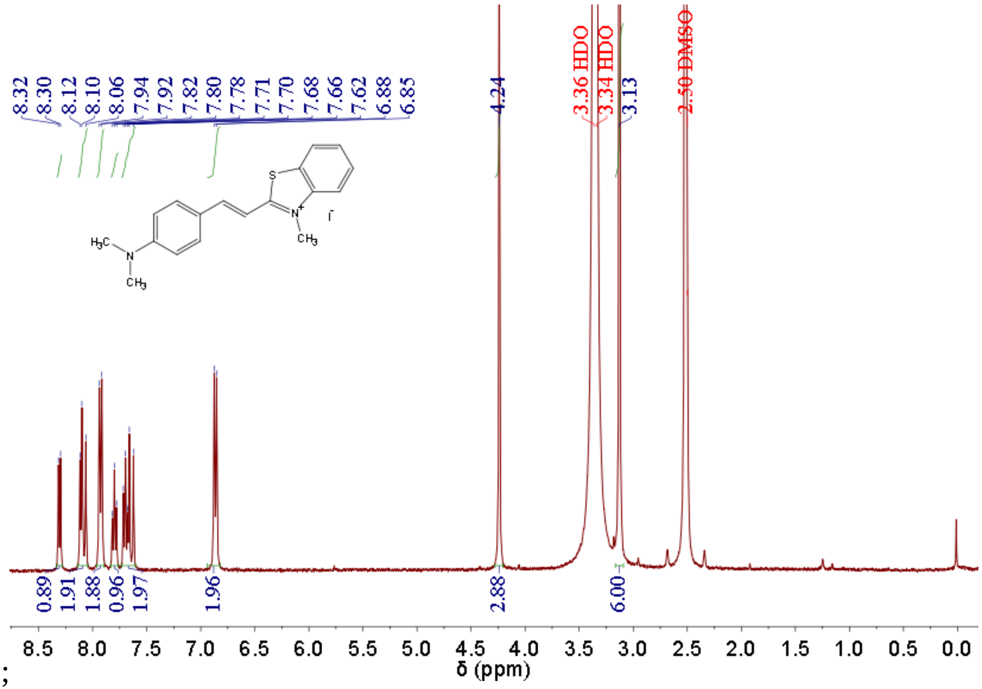

;

Figure S28. The 1H NMR of compound SZI-1.

Acquisition Parameter			
Acquisition Mode	Single MS	Acquired Scans	4
Polarity	Positive		
Broadband Low Mass	57.7 m/z	Broadband High Mass	500.0 m/z

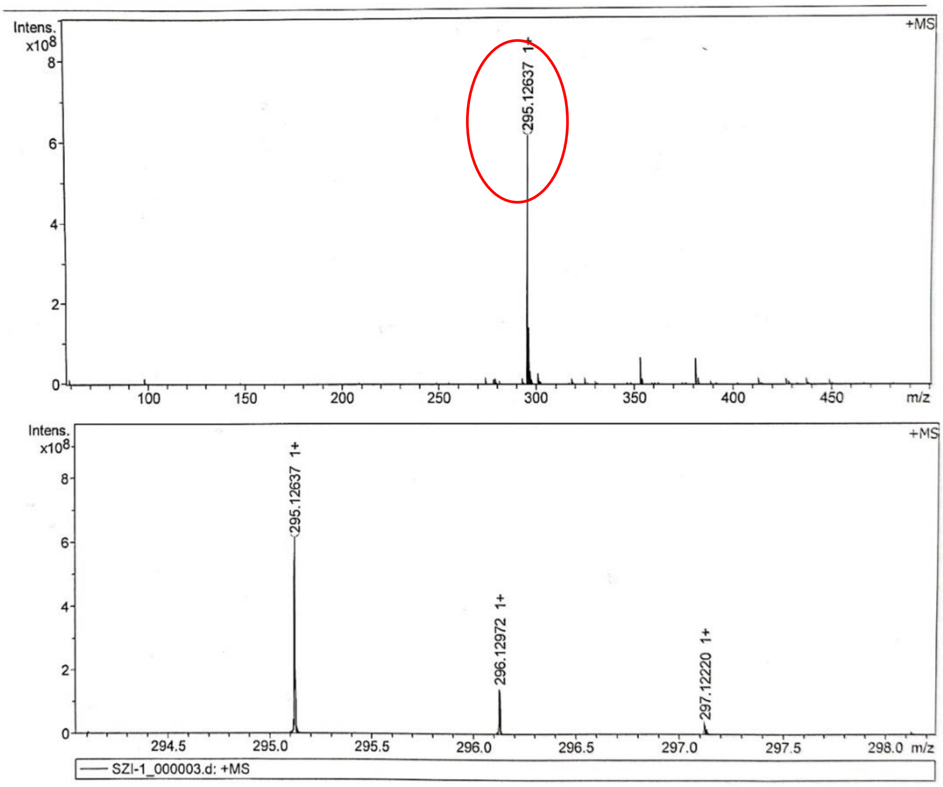

Meas. m/z	#	Ion Formula	Score	m/z	err [ppm]	Mean err [ppm]	mSigma	rdb	e⁻ Conf	N-Rule
295.126366	1	C18H19N2S	100.00	295.126346	-0.1	-0.2	34.2	10.5	even	ok

Figure S29. Mass Spectra (ESI) of SZI-1

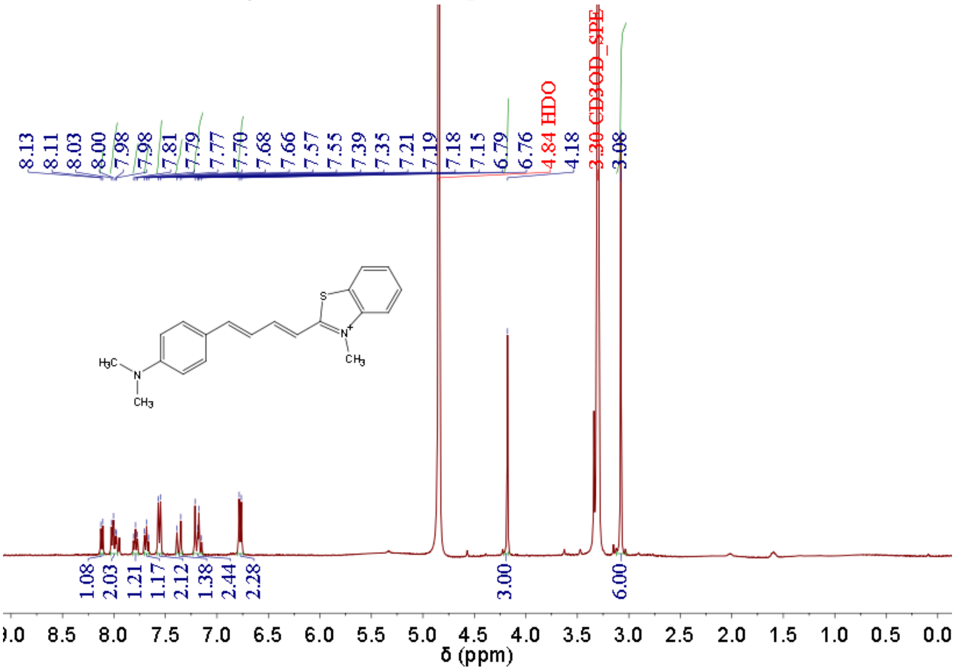

Figure S30. The ^{1}H NMR of compound SZI-2.

Acquisition Parameter

Acquisition Mode	Single MS	Acquired Scans	20
Polarity	Positive	Source Accumulation	0.001 sec
Broadband High Mass	500.0 m/z	Ion Accumulation Time	0.050 sec

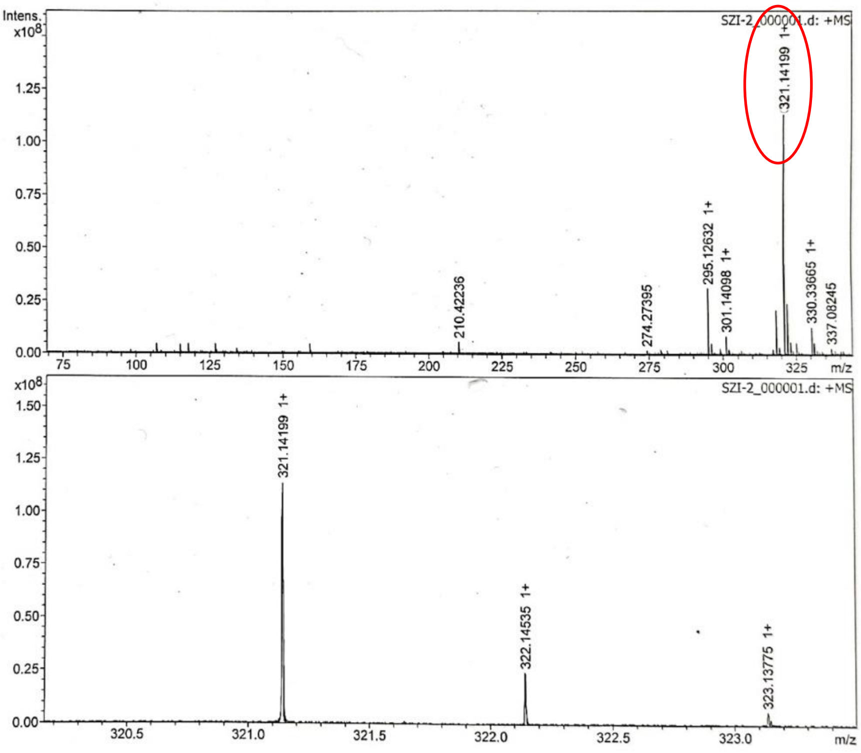

Meas. m/z	#	Ion Formula	Score	m/z	err [ppm]	Mean err [ppm]	mSigma	rdb	e⁻ Conf	N-Rule
321.141991	1	C20H21N2S	100.00	321.141996	-0.0	-0.1	36.7	11.5	even	ok

Figure S31. Mass Spectra (ESI) of SZI-2

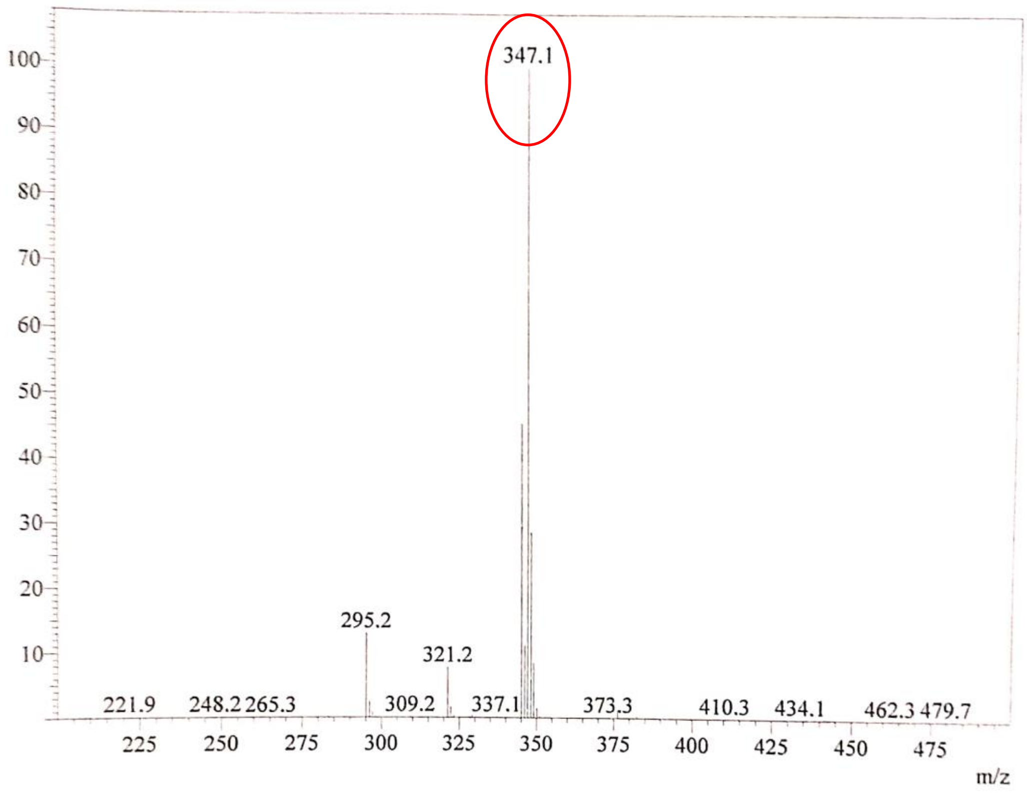

Figure S32. Mass Spectra (ESI) of SZI-3

References

(1) Cao, K.; Farahi, M.; Dakanali, M.; Chang, W. M.; Sigurdson, C. J.; Theodorakis, E. A.; Yang, J. *J. Am. Chem. Soc.* **2012**, 134, 42, 17338–17341.

(2) Diner, I.; Dooyema J.; Gearing, M.; Walker, L. C.; Seyfried, N. T. *Bioconjugate Chemistry* **2017**, 28, 10, 2627-2637.

(3) Fu, W.; Yan, C.; Guo, Z.; Zhang, J.; Zhang, H.; He, T.; Zhu, W. *J. Am. Chem. Soc.* **2019**, 141, 7, 3171–3177.

(4) Li, M.; Zhao, A.; Ren, J.; Qu, X. *ACS Chem. Neurosci.* **2017**, 8, 6, 1299–1304.

(5) Lv, G.; Sun, A.; Wei, P.; Zhang, N.; Lan H.; Yi T. *Chem. Commun.* **2016**, 52, 8865.

(6) Li, Y.; Xu, D.; Sun, A.; Ho, S. L.; Poon, C. Y.; Chan, H. N.; Ng, O. T. W.; Yung, K. K. L.; Yan, H.; Li, H. W.; Wong, M. S. *Chem. Sci.* **2017**, 8, 8279-8284.

(7) Guan, Y.; Cao, K. J.; Cantlon, A.; Elbel, K.; Theodorakis, E. A.; Walsh, D. M.; Yang, J.; Shah, J. V. *ACS Chem. Neurosci.* **2015**, 6, 9, 1503–1508.

(8) Jung, S. J.; Lee, J. Y.; Kim, T. H.; Lee, D. E.; Jeon, J.; Yang, S. D.; Hur, M. G.; Min, J. J.; Park, Y. D. *Bioorg. Med. Chem. Lett.* **2016**, 26, 7, 1784-1788.
